# A Genome-Wide Analysis of Long Noncoding RNAs in Circulating Leukocytes and Their Differential Expression in Type 1 Diabetes Patients

**DOI:** 10.1155/2020/9010314

**Published:** 2020-11-25

**Authors:** Yihan Liu, Xiaoming Du, Jia Cui, Changlong Li, Meng Guo, Jianyi Lv, Xin Liu, Jingtao Dou, Xiaoyan Du, Hongjuan Fang, Zhenwen Chen

**Affiliations:** ^1^School of Basic Medical Sciences, Capital Medical University, Beijing 100069, China; ^2^Institute of Acupuncture and Moxibustion, China Academy of Chinese Medical Sciences, Beijing 100700, China; ^3^Tianjin Stomatological Hospital, Tianjin Key Laboratory of Oral Function Reconstruction, Hospital of Stomatology, Nankai University, Tianjin 300041, China; ^4^Department of Endocrinology, Chinese PLA General Hospital, Beijing 100853, China; ^5^Department of Endocrinology, Beijing Tiantan Hospital, Capital Medical University, Beijing 100070, China

## Abstract

Long noncoding RNAs (lncRNAs) regulate gene expression at different levels in various diseases, including type 1 diabetes (T1D). However, the expression of circulating lncRNAs in leukocytes in T1D has not been well documented. To identify differentially expressed lncRNAs between T1D patients and healthy controls, RNA sequencing was performed on samples of leukocytes collected from both healthy persons and T1D patients. The categories, enriched pathways, coexpression networks, and the characteristics of novel lncRNAs were analyzed to provide an extensive profile. qPCR was adopted to validate the differential expression of lncRNAs in the validation cohort. A total of 14,930 lncRNAs and 16,063 mRNAs were identified in the peripheral blood leukocyte of T1D patients. After optimization using an adjusted *p* value (threshold of <0.05), 393 circulating lncRNAs were identified, of which 69 were downregulated and 324 were upregulated in T1D patients. Gene Ontology analysis indicated that these lncRNAs and mRNAs were enriched in the immune system category. Further analysis showed that 61.28% of the novel lncRNAs were conserved in humans. A set of 12 lncRNAs were selected for qPCR validation, and 9 of 12 lncRNAs were confirmed to show significant differential expression between the T1D and control validation cohorts. Among the 9 confirmed lncRNAs, lncRNA MSTRG.128697 and lncRNA MSTRG.128958 were novel and human-specific; however, further validation is required. lncRNA MSTRG.63013 has orthologous sequences in the mouse genome and was identified as a key node for etiology and pathophysiology in animal studies, which will help understand the epigenetic mechanisms of T1D complications.

## 1. Introduction

Type 1 diabetes (T1D) is defined as an autoimmune disease resulting from a combination of environmental and genetic factors [1, 2]. As the worldwide prevalence of T1D is increasing, the burden of morbidity and mortality associated with the concomitant microvascular and macrovascular complications is rising as well [3, 4]. In China, the estimated prevalence of T1D is 1.01 per 100,000 persons/year for all age groups [5]. Compared with type 2 diabetes patients, T1D patients are exposed to a higher risk of all-cause mortality and cardiovascular disease [6]. Literature and practice show that the therapeutic efficacy for T1D patients could be improved with early detection and timely intervention.

The pathogenesis of T1D has three stages: asymptomatic beta cell autoimmunity with normoglycemia, asymptomatic beta cell autoimmunity with dysglycemia, and overt T1D [7]. Prospective studies showed that the autoimmune antibodies precede and predict the onset of T1D, which appears earlier than that of dysglycemia [8]. An early diagnosis of T1D is not always feasible. Autoimmune antibodies are generally regarded as a biomarker for T1D; however, they are not sufficiently specific and sensitive to meet the diagnostic requirements [9]. Therefore, further investigation of potential T1D diagnostic markers is urgently required.

Long noncoding RNAs (lncRNAs) are a type of RNA transcripts that have emerged as crucial regulators of gene expression in various pathophysiological conditions [10–13]. lncRNAs have demonstrated noteworthy versatility, as they wield their functions through interactions with RNA, DNA, or proteins [14]. Accumulating data have emphasized the important role of lncRNAs in many inflammatory and autoimmune diseases, including T1D [14]. Abnormal lncRNAs may cause autoimmune reactions and alter the progression of T1D and its associated complications [15, 16]. To the best of our knowledge, effective diagnosis, prevention, and treatment methods for T1D have not yet been well established. Further studies on the relationship between lncRNA and T1D will provide new targets for both diagnosis and treatment of T1D.

There are only a few recent studies on selecting and identifying new lncRNAs in T1D, merely covering the function of lncRNA in *β* cells in a T1D mouse model [17, 18]. However, research into the identification of lncRNAs in T1D as an autoimmune disease, especially focusing on circulating lncRNAs, is limited. It has been reported that approximately 50% of the genetic risks for T1D are known to reside within the human leukocyte antigen (HLA) region [14]. We hypothesized that T1D, as an autoimmune disease, has some critical lncRNAs in leukocytes that could be potential biomarkers or regulators of the disease. Moreover, these lncRNAs could be transferred with or secreted in extracellular vesicles (such as exosomes) that would then circulate to other parts of the body and could have a regulatory role in T1D [19].

Therefore, in the current study, we performed whole genome RNA sequencing to identify the transcriptome profile of leukocytes extracted from the blood of T1D patients and to examine the differential expression of lncRNAs between T1D patients and healthy controls. The investigation into the correlation of circulating lncRNAs and T1D may lead to a broader understanding of pathogenesis and stimulate new ideas for diagnosis and prognosis of T1D.

## 2. Materials and Methods

### 2.1. Subjects

Human blood samples were collected from the Department of Endocrinology, Chinese PLA General Hospital, and separated into the following groups: healthy controls (CTL, *n* = 6) and T1D patients (T1D, *n* = 6) as the discovery cohort by clinical examination. The validation cohort was grouped as healthy controls (CTL, *n* = 36) or T1D patients (T1D, *n* = 23). All subjects in the study were enrolled between March 2017 and January 2018. Blood samples were subsequently collected following an overnight fast of 10 to 12 h. The study was approved by the Ethics Committee of the Chinese PLA General Hospital (Permitted No. S2016-147-03), and all patients provided informed consent.

### 2.2. Detailed Information of Cohorts

For both discovery and validation cohorts, the following subjects were included in the T1D group: (1) diagnosed with T1D according to the World Health Organization (WHO) screening criteria, including the 75 g oral glucose tolerance test, C-peptide testing, and autoantibody test (glutamic acid decarboxylase, islet cell antibody, and insulin antibody); (2) aged 18-65 years without any gender bias; and (3) free from all endocrine diseases and administered no other drugs except insulin. The subjects in the healthy control group were (1) healthy with a negative diagnosis of T1D as defined by the World Health Organization (WHO) and with normal blood biochemical indexes; (2) free from all endocrine disease; and (3) aged 18-65 years old without a gender bias. Exclusion criteria were (1) current or previous severe disease or tumors in the heart, brain, liver, or kidney; (2) severe gastrointestinal diseases; (3) presence of other conditions, such as severe infection or active tuberculosis with multiple antibiotics used; (4) pregnancy or lactation; (5) a history of current alcohol and/or drug abuse; (6) a history of mental illness or family history thereof; or (7) stressful event occurring within the past year.

### 2.3. Total RNA Extraction and Purification from Leukocytes

Total RNA was extracted from the leukocytes isolated from peripheral blood. Briefly, approximately 3.5 mL of blood from each subject was incubated at room temperature for less than 4 h, followed by centrifugation for 10 min at 3000 × *g*. The cell pellet was then incubated with 1 mL red blood cell lysis buffer, and the resultant lysate was centrifuged for 3 min at 3000 × *g*. Total RNA was isolated from purified leukocyte pellet using the RNeasy kit (Qiagen Inc., Valencia, Calif., #217061). Ribosomal RNA was removed using the Ribo Zero Magnetic Gold kit (MRZG126, Illumina). Quality and integrity of the isolated RNA was verified by NanoDrop 2000c (Thermo Fisher Scientific, Waltham, MA, USA) and Bioanalyzer (Agilent Technologies Inc., USA). OD260/280 ratio ranged between 2.0 and 2.2 and RIN > 7.0.

### 2.4. RNA Sequencing and Analysis

RNA sequencing was performed by Annoroad Gene Tech. Co., Ltd. Sequence libraries were prepared using the Illumina TruSeq Stranded mRNA LT kit without poly(A) selection in order to include all the lncRNA transcripts that were not polyadenylated. Libraries were sequenced on the Illumina HiSeq X Ten with 150 bp paired-end reads, and each sample obtained 10 Gbp. The paired-end reads from the samples were mapped to the hg19 reference genome by HISAT2. Ab initio transcript reconstruction was performed using StringTie, version 1.3.2d, with the reference genome obtained from ENSEMBL. Novel transcripts having at least 2 exons were included. Read counts were then calculated per transcript from the alignment bam files using HTSeq (v 0.6.0). Transcripts with minimal expression (mean counts across all conditions) were filtered out. The protein-coding potential of transcripts was evaluated using the CNCI, CPC, PFAM, and CPAT analysis. Novel lncRNAs were identified as noncoding RNA in all four analyses. Conservative analysis of the identified novel lncRNAs was performed by PhastCons. Differentially expressed (DE) noncoding transcripts were detected using DESeq. A negative binomial distribution statistical method was used to standardize the data, and obtained *p* values were subjected to multiple tests to correct for false positives according to the Benjamini and Hochberg methods. Empirical Bayes moderated statistics and corresponding *p* values were computed for comparisons, and *p* values were adjusted for multiple comparisons using the Benjamini-Hochberg procedure. Transcripts with an adjusted *p* value of <0.05 were considered differentially expressed and defined as optimized data.

### 2.5. Gene Ontology (GO) Term Analysis

The GO term network was constructed on the basis of similarities among GO terms globally. The terms were supplied as annotation to genes and gene products. In this study, we mainly focused on the biological process (BP), cellular component (CC), and molecular function (MF) domains. Calculations of pathway enrichments were used on optimized data. We identified mRNAs within 50 kb lncRNAs and calculated the correlation between DE-lncRNAs and mRNAs based on Pearson's correlation greater than 0.90 as the target mRNA of lncRNA.

### 2.6. Construction of the Coding/Noncoding Gene Coexpression Network

To explore the association between lncRNAs and target mRNAs, a coding/noncoding coexpression (CNC) network was constructed based on the correlation analysis between DE-lncRNAs and mRNAs. For each pair of analyzed transcripts, Pearson's correlation was calculated, and pairs with significant correlations (0.90 or greater) were used to construct a network using Cytoscape (http://www.cytoscape.org) and STRING (https://string-db.org). The network was visualized using the open-source bioinformatics software Cytoscape. Each transcript corresponded to a node, and the connection of two transcripts was represented by an edge, indicating significant correlation.

### 2.7. Quantitative Real-Time PCR for Validation of Differential lncRNA

A total of 15 lncRNAs were chosen to be validated further by real-time PCR in independently expanding samples of CTL (*n* = 36) and T1D (*n* = 23), respectively. These 15 candidate lncRNAs were chosen based on the following criteria: (1) they were the top 10 DE-lncRNAs between T1D and controls; (2) the biotypes of both the lncRNA and antisense-lncRNA were included; (3) lncRNAs with no significant differential expression between T1D and control were randomly selected; (4) both novel and known lncRNAs were included; (5) both up- and downregulated DE-lncRNAs were included; and (6) the lncRNAs in the CNC network were chosen. After the primer design and optimization of PCR conditions, 12 lncRNAs were selected for testing, and the information is presented in Supplementary Table S1.

Total RNA from CTL (*n* = 36) and T1D (*n* = 23) samples was extracted using TRIzol, as per the manufacturer's instructions (Invitrogen). Double-stranded cDNA was reverse-transcribed by 5X All-In-One MasterMix (AccuRT Genomic DNA Removal Kit; Applied Biological Materials Inc., Canada) according to the manufacturer's instructions. The quantitative real-time PCR (qPCR) was performed using EvaGreen 2X qPCR MasterMix-No Dye (SYBR Green; Applied Biological Materials Inc., Canada), and samples were amplified using the CFX Connect qPCR System (Bio-Rad, Hercules, CA, USA). All experiments were conducted in triplicate and replicated three times. The 2^-*ΔΔ*CT^ method was used to quantify the relative expression of each lncRNA, with *β*-actin as an internal control.

### 2.8. Statistical Analysis

Statistical analysis was performed using SPSS 19.0 software (SPSS Inc., USA). All values were expressed as the mean ± SEM. Comparisons between groups were made using Student's *t*-test or one-way ANOVA. Differences were considered statistically significant at *p* < 0.05. Different expression levels of lncRNAs in the expanding samples were evaluated with the Mann–Whitney *U* test. Differences were considered statistically significant at *p* < 0.05.

## 3. Results

### 3.1. Transcriptomic Landscape of T1D Patients

**Figure 1:**
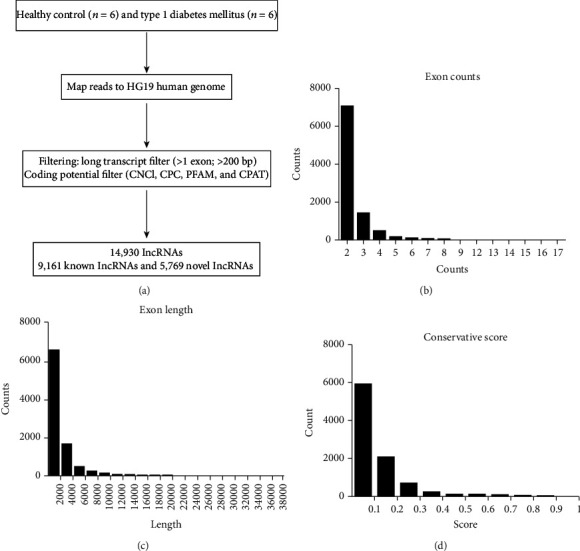
Transcriptomic landscape of T1D lncRNAs. (a) A schematic illustration of the procedure used to identify and define lncRNAs in the leukocytes of controls and T1D patients. (b) The transcripts of novel lncRNAs were mainly distributed in 2 exons, 3 exons, and 4 exons. (c) The largest number of novel lncRNA transcripts was concentrated at a length less than 2500 nt. (d) The transcripts of novel lncRNAs were concentrated at the lowest conservation score range.

Transcriptomic profiles to identify critical transcripts related to T1D were investigated in 6 patients diagnosed with T1D and 6 healthy controls (process flow chart shown in Figure 1(a)). Information about these T1D participants and expansion cohort patients are shown in Table 1. In order to thoroughly investigate the T1D transcriptome, we analyzed 14,930 detected lncRNAs in the leukocytes of patients with T1D compared with those of healthy controls. There were 9161 lncRNAs already registered in the databases (defined as known), of which 4983 were upregulated and 4178 were downregulated. Meanwhile, 5769 lncRNAs were identified for the first time here (defined as novel), with 3857 and 1912 lncRNAs up- and downregulated, respectively. Known lncRNAs could be classified into long intervening noncoding (lincRNA) (40.68%), antisense (38.94%), sense intronic (8.06%), to be experimentally confirmed (TEC; 6.82%), processed transcripts (3.80%), sense overlapping (1.48%), and others (0.22%). Novel lncRNAs were only categorized as either antisense (2473; 42.87%) or intronic (3296; 57.13%). We also identified 16,063 differentially expressed mRNAs (T1D-mRNAs; 8620 downregulated and 7443 upregulated) between T1D and healthy controls. Data can be seen in the GEO with accession number GSE130279.

The characteristics of 5769 novel lncRNAs identified in T1D patients versus healthy controls were analyzed further. Most novel lncRNA transcripts harbored 2 exons (6538/9083, 71.98%) and second to that is lncRNA transcripts with 3 exons (1358/9083, 14.95%; Figure 1(b)). The exon length was less than 2000 bp in most of the novel lncRNAs and was never longer than 12,000 bp (Figure 1(c)). We analyzed the conservation of the novel lncRNAs in humans (Figure 1(d)) and found that the percentage of transcripts with conservation scores (CS) less than 0.1 was 61.28%. Only 3.88% of the novel lncRNA transcripts (352/9083) had a CS of zero, which meant absolute conservation in humans. Analysis of the distribution of transcripts on chromosomes demonstrated that novel lncRNAs were mainly found on chr1, chr2, chr3, chr4, chr5, and chr6, and most of lncRNAs that exhibited low CS in humans were from the same chromosome (Supplementary Figure S1).

### 3.2. Differentially Expressed lncRNAs and mRNA between T1D and Healthy Controls

**Figure 2:**
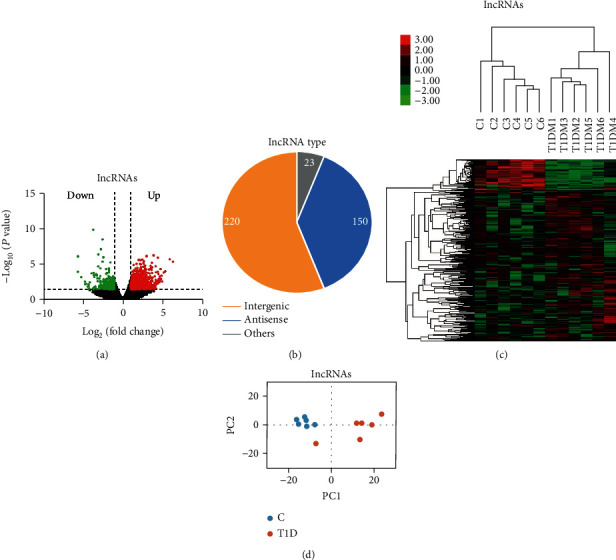
Features of novel lncRNAs in T1D patients as compared with those in healthy controls. (a) Differentially expressed lncRNAs (T1D-lncRNAs) were identified from a volcano plot showing data from T1D patients relative to healthy controls. The vertical black lines correspond to twofold up- and downregulation, respectively, and the horizontal black line represents a *p* value of 0.05. The red and green points in the plots represent the differentially expressed transcripts in T1D patients with statistical significance for upregulation (324 lncRNAs) and downregulation (69 lncRNAs), respectively. (b) Pie chart representations show the proportion of T1D patients associated with lncRNAs that are transcribed as antisense (blue), intergenic (orange), or other types (grey) and analyzed postoptimization. (c) Differential lncRNA expression profiles were hierarchically cluster analyzed and shown as a heat map, wherein 393 lncRNAs were upregulated (red) or downregulated (green). (d) Principal component analysis results.

Next, we optimized these lncRNAs according to the following criteria: (1) at least one sample had to display expression of a given lncRNA in each group; (2) fold change had to be >2 between T1D and control; and (3) statistical testing must result in *p* adjusted < 0.05. After optimization, we identified 393 DE-T1D-lncRNAs (69 downregulated and 324 upregulated), in which 150 were antisense, 220 were intergenic (lincRNA), and 23 belonged to other subtypes (Figures 2(a) and 2(b), Supplementary Table S1). The top 20 lncRNAs based on their abundance are listed with their sequencing data in Table 2. T1D-lncRNAs were then clearly distinguished by hierarchical clustering (Figure 2(c)) and principal content analysis (Figure 2(d)).

**Figure 3:**
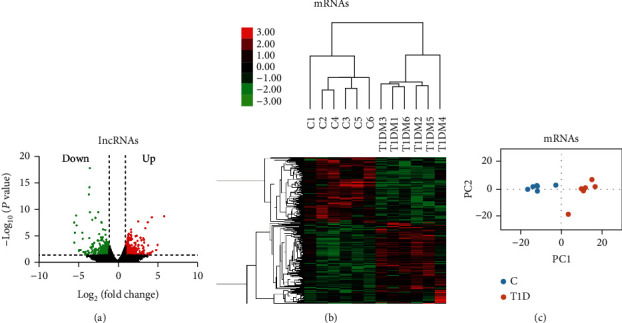
Transcriptomic landscape of T1D-mRNAs. (a) T1D differentially expressed mRNAs (T1D-mRNAs) were identified from the volcano plot in T1D patients relative to normal controls. The vertical black lines correspond to twofold up- and downregulation, respectively, and the horizontal black line represents a *p* value of 0.05. The red and green points in the plots represent differentially expressed transcripts with statistical significance for upregulation (172 mRNAs) and downregulation (139 mRNAs), respectively. (b) Differential mRNA expression profiles were hierarchically cluster analyzed and shown as heat maps, wherein upregulated genes are depicted in red and downregulated genes are depicted in green. (c) Principal component analysis also showed that mRNAs are distinguishable between T1D patients and controls.

The T1D-mRNAs were also optimized, and a total of 311 mRNAs were different in T1D patients compared to those in the healthy controls, with 172 upregulated and 139 downregulated (Figure 3(a), Supplementary Table S2). Hierarchical cluster analysis (Figure 3(b)) and principal content analysis (Figure 3(c)) showed similar results and exhibited the same trends as the DE-lncRNAs results. Both DE-lncRNA and DE-mRNA were distinguishable in T1D patients compared with healthy controls by hierarchical clustering and principal content analysis. The number of optimized T1D-mRNAs was less than that of T1D-lncRNAs. Taken together, the different expression levels of protein-coding genes and hundreds of lncRNAs in leukocytes presented transcriptional differences between T1D patients and healthy controls.

### 3.3. The Functions of Differentially Expressed lncRNA and mRNA

**Figure 4:**
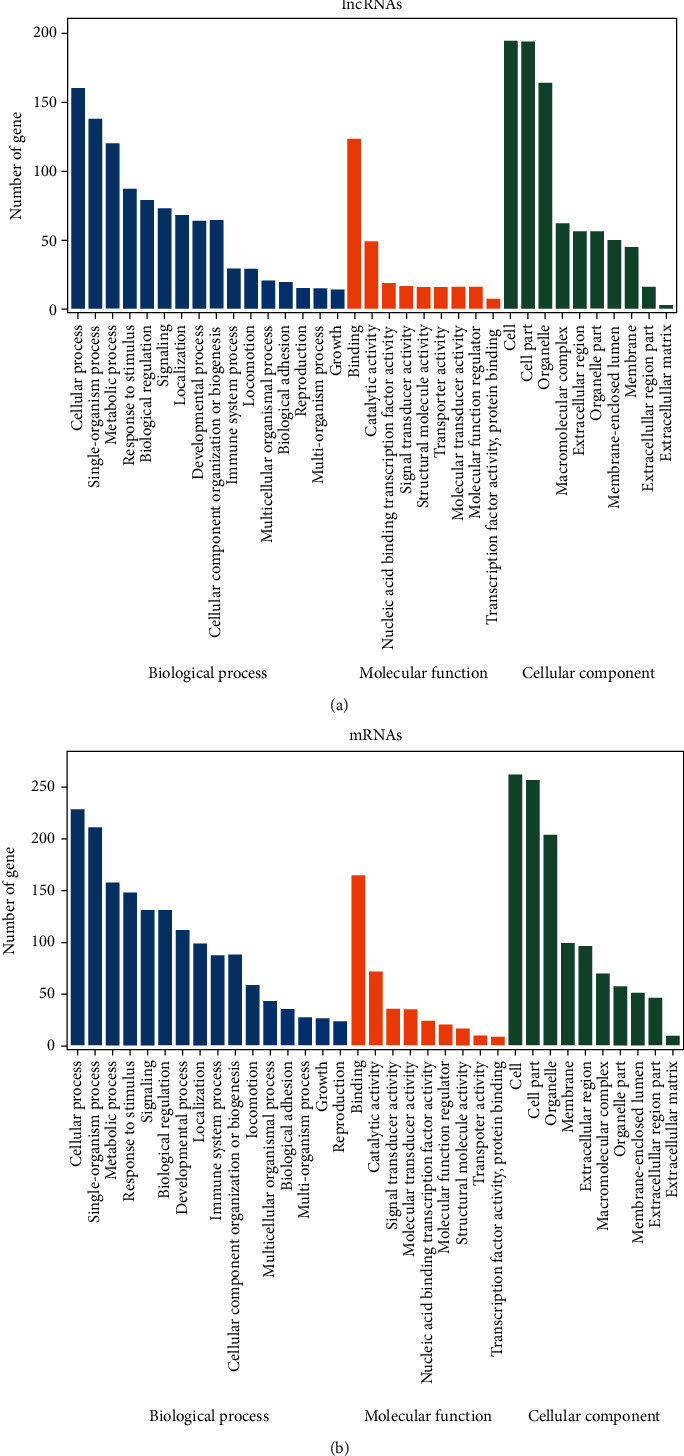
Bioinformatics analysis of T1D-lncRNA and T1D-mRNA. Gene Ontology analysis of T1D-lncRNAs (a) and T1D-mRNAs (b) in biological processes, molecular functions, and cellular components.

We applied GO enrichment analysis to classify the optimized T1D-lncRNAs and T1D-mRNAs based on three main categories, namely, biological process, molecular function, and cellular component (Figures 4(a) and 4(b)). In the biological process category, we found that both dysregulated DE-lncRNA and DE-mRNAs were enriched in 16 items under the umbrella of biological processes (BPs). These included cellular processes, single organism processes, metabolic processes, responses to stimuli, biological regulation, localization, developmental processes, cellular component organization or biogenesis, immune system processes, or locomotion (Figures 4(a) and 4(b)). Under the molecular function (MF) category, binding, catalytic activity, nucleic acid binding transcription factor activity, signal transducer activity, and structural molecule activity were in the top 5 percent (Figures 4(a) and 4(b)). Both DE-lncRNAs and DE-mRNAs may be enriched in the immune system category and the metabolic process category (the 3^rd^ BP term). When we further analyzed the top 20 items in the categories, we found biological processes, molecular functions, and cellular content pathways; metabolism-related terms were not present (Supplementary Figure S2 and S3).

**Figure 5:**
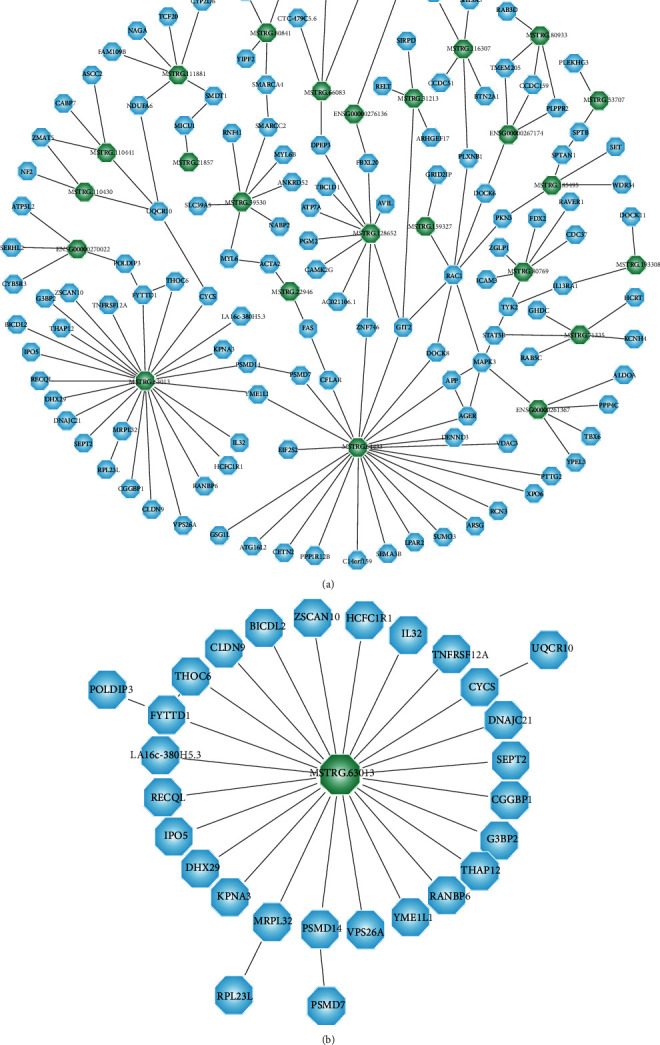
The construction and analysis of a gene coexpression regulation network. (a) Coexpression networks of lncRNA-mRNA derived from 156 network nodes and (b) the network of key lncRNA MSTRG.63013.

Genes with the same biological function or regulating the same pathway have similar expression patterns. Thus, a coexpression network may provide information about the function of lncRNAs and could be used to predict lncRNA function. We built a lncRNA-mRNA coexpression network with DE-lncRNAs. There were 24 lncRNAs and 138 mRNAs found in CNC network analysis, which were derived from 156 network nodes (Figure 5(a)).

### 3.4. Measurement of Chosen lncRNAs in the Validation Groups

**Figure 6:**
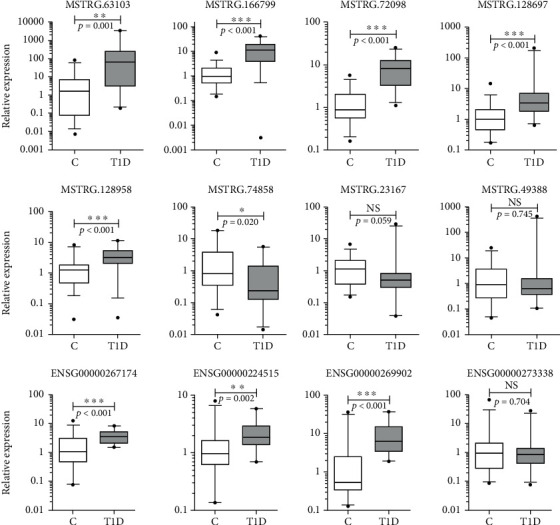
Results of 12 lncRNAs confirmed in the validation cohort by qPCR. There are 9 lncRNAs that showed significant differences in expression levels between T1D patients (*n* = 23) and healthy controls (*n* = 36). NS: no significant difference. ∗ indicates significant difference with *p* < 0.05 and ∗∗ indicates *p* < 0.01.

**Figure 7:**
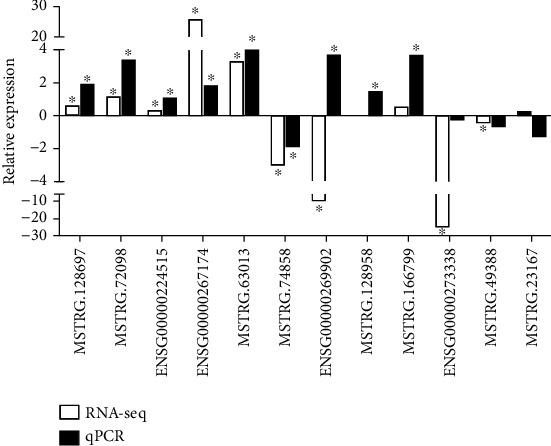
Comparisons of the results between RNA sequencing data and confirmation of 12 lncRNAs in the validation cohorts by qPCR. ∗ indicates significant difference in RNA sequencing data or validation cohorts by qPCR between T1D patients and healthy controls.

To further confirm the lncRNAs differentially expressed in T1D, we performed independent measurement of 12 lncRNAs in the validation groups which consisted of T1D patients (*n* = 23) and healthy controls (*n* = 36) using real-time PCR. The expression levels of lncRNAs in the validation cohort are shown in Figure 6. Here, 9 of 12 (75%) lncRNAs exhibited significant differential expression levels between the T1D and control groups. These included lncRNAs MSTRG.128697, MSTRG.128958, MSTRG.74858, MSTRG.72098, MSTRG.63013, MSTRG.166799, ENSG00000224515, ENSG00000269902, and ENSG00000267174; of these, MSTRG.74858 was downregulated and the others were all upregulated in T1D. In addition, lncRNAs MSTRG.128697, MSTRG.74858, MSTRG.72098, MSTRG.63013, and ENSG00000269902 belonged to the lincRNA biotype, while MSTRG.166799 and ENSG00000224515 belonged to the antisense lncRNA biotype. The known lncRNA ENSG00000267174 is a 3′ overlapping lncRNA. We then compared the validation results and sequencing data and found that most lncRNAs in the expanded group displayed similar trends with the sequencing data (Figure 7). In particular, six lncRNAs, namely, MSTRG.128697, MSTRG.72098, ENSG00000224515, ENSG00000267174, MSTRG.74858, and MSTRG.63013, exhibited exact results as in the sequencing data.

We also sought to identify potential orthologs of the nine lncRNAs by comparing their sequences with previously identified murine lncRNAs. Based on pairwise genomic alignments, we found that four lncRNA sequences (44.5%) harbored orthologs in mouse genomic sequences without annotation, whereas the other five lncRNAs were unique to humans, with no orthologs in mice (Table 3). lncRNA MSTRG.128697 and lncRNA MSTRG.128958 were defined as novel and human-specific, which makes them worthy of further investigation in relation to human T1D epigenetic mechanisms or as biomarkers.

In terms of predicting lncRNA function in the coexpression network, 4 of 9 validated lncRNAs were predicted to have mRNA targets related to 32 genes (Table 4). A total of 16 genes were included in the network of MSTRG.63013, with a correlation score over 0.9, and there were 8 genes within 50 kb of MSTRG.63013 (Figure 5(b) and Supplementary Table S3). lncRNA MSTRG.63013 exhibited orthologous sequences in the mouse genome, which has been identified as a key node in the etiology and pathophysiology in animal studies for the development of T1D.

## 4. Discussion

lncRNAs are involved in a variety of biological functions and pathophysiological mechanisms underlying diabetes. However, the lncRNA profile in leukocytes and the differential expression of lncRNAs between T1D patients and healthy controls is currently unknown. To the best of our knowledge, we are pioneers in constructing full profiles of circulating leukocytic lncRNA and mRNA in T1D patients. In addition, a set of 9 lncRNAs was confirmed and validated to have significant differential expression between T1D patients and controls.

lncRNAs participate in the epigenetic regulation of a variety of diseases by altering the expression of lncRNA target genes and display clear clinical significance [20]. Increasing studies showed that diabetic susceptibility loci are associated with abnormal expression of lncRNAs [21]. Recent studies have focused on the identification of new lncRNAs and their functions in blood or immune cells in immune-related diseases [22]. They have demonstrated that lncRNAs play distinct roles in modulating immune cell activation, especially in human autoimmune diseases. Gagliardi et al. characterized the action of lncRNAs in peripheral blood mononuclear cells from amyotrophic lateral sclerosis patients [23]. Aune et al. identified lncRNAs differentially expressed in whole blood from patients of various autoimmune diseases and even found that novel lncRNA loci were localized near leukocyte transcriptional enhancers instead and not randomly distributed across the genome [24]. Hence, we reasoned that lncRNAs might alternatively play a regulatory role in such peripheral blood cells as leukocytes and, in turn, alter cellular phenotypes and play an active role in T1D.

Studies investigating the link between lncRNAs and the development of diabetes have only recently been undertaken [25]. Although the characteristics of T1D are well known, epigenetic mechanisms, such as the function of noncoding RNAs, have mainly focused on pancreatic *β* cell disorders and insulin resistance, with few reports on relation with the immune system [14]. As an autoimmune disease, the susceptibility gene located in the HLA region is by far the greatest contributor to the development of T1D [26]. This warrants the need to screen autoimmune-related lncRNAs in peripheric blood for T1D. Our results indicated that T1D leukocytes harbor enriched lncRNAs and may represent an important target for further diabetes research. The most obvious limitations of this study were the small size of our cohort and its single-institution design. Optimally, a larger series is needed to validate these candidate lncRNAs.

To further confirm and understand lncRNAs in T1D, we validated 12 lncRNAs in the expansion group. Nine lncRNAs (9/12, 75%) were confirmed to have significant differential expression between T1D patients and healthy controls. The identification rate we described here is comparable or higher than the identification rate of lncRNAs in whole blood samples from sclerosis patients (5/7, 71.42%) [27] or in blood samples from diabetic neuropathy patients (2/6, 33.33%) [28]. We noticed that the positive validation ratio of lncRNAs in the top 10 DE-lncRNAs in sequencing cohorts was lower in lncRNAs without significant expression in sequencing cohorts and that there were lncRNAs without expression in either group of sequencing cohorts. These results indicate that candidate lncRNAs selected for validation broaden the chosen range, without being limited to only the topmost differentially expressed lncRNAs in sequencing cohorts. In the future, we plan to screen lncRNAs in circulating exosomes and compare them with the lncRNAs identified in leukocytes from the current study.

lncRNAs are low in abundance, are mostly spliced with few exons, and have tight tissue specificity [29, 30], emphasizing the importance of studies on human-specific lncRNAs in human physiology and diseases. In the current study, we analyzed the conservation of novel lncRNAs and nine specifically validated lncRNAs. For the novel lncRNAs, more than 60% of the lncRNAs had a conservation score of less than 0.1, meaning they exhibited high conservation in humans. For the set of validated 9 lncRNAs, 2 lncRNAs were novel and human-specific but had no further annotation. Thus, these 2 lncRNAs could be used directly in clinical research without any further need to consider the conservation problem. It is important to investigate human data with samples from humans or humanized models [31]. Therefore, the large amounts of data on lncRNAs and mRNAs that we generated from blood samples of T1D patients represent a useful and valuable contribution, based on an urgent need for human data.

Four of the nine validated lncRNAs had predicted mRNA targets related to 32 genes, of which *SPOP* [32] and *DOCK6* [33] have been shown to be associated with diabetes. The other 5 lncRNAs lacked predicted mRNA targets, which may arise from our currently limited understanding of the human genome (or that of other animals). Intriguingly, the lncRNA MSTRG.63013 was a key node in the coexpression analysis. There are 16 genes associated with this lncRNA and 8 genes within 50 kb of it, especially *G3BP2* [34] and *CYCS* [35], which are involved in many cell signaling pathways and RNA metabolism. *IL32*, a major autoimmune member of *β* cells in children [36], is also included in the list. In addition, the other genes listed include *PSMD14* and *TNFRSF12A*, which are related to key targets in the TNF pathway of autoimmune diseases. lncRNA MSTRG.63013, which exhibited orthologous sequences in the mouse genome, could be a key node of T1D etiology and pathophysiology in animal studies.

In conclusion, the leukocyte specificity observed for the nine lncRNAs identified here (together with other data presented here) could be conducive to the development of lncRNA-based diagnosis and treatment for T1D. lncRNA MSTRG.128697 and lncRNA MSTRG.128958 were novel and human-specific and may be useful as early diagnostic markers for T1D in clinical practice. lncRNA MSTRG.63013 could be used in animal experiments, which might accelerate research on the epigenetic mechanism of T1D.

## Figures and Tables

**Table 1:** Clinical characteristics of type 1 diabetes (T1D) patients and healthy controls in the discovery and validation cohorts.

Items	Type 1 diabetes in discovery cohort (*n* = 6)	Type 1 diabetes in validation cohort (*n* = 23)		Healthy controls in discovery cohort (*n* = 6)	Healthy controls in validation cohort (*n* = 36)
Age (years)	30.00 ± 15.55	32.45 ± 15.39		32.5 ± 9.8	28.3 ± 6.4
Sex (male, %)	33.33%	26.09%		33.33%	33.33%
BMI (kg/m^2^)	20.77 ± 1.51	21.93 ± 3.10		19.7 ± 1.0	20.1 ± 1.2
Fasting glucose (mmol/L)	11.57 ± 4.47	9.49 ± 4.80		4.8 ± 0.6	4.8 ± 0.5
Fasting insulin (*μ*U/mL)	10.13 ± 2.61	4.22 ± 5.78		11.0 ± 0.7	13.2 ± 3.6
Fasting C-peptide (ng/mL)	0.28 ± 0.23	0.46 ± 0.70		1.9 ± 0.7	2.1 ± 0.5
HbA1c	8.95 ± 1.97	8.67 ± 2.40		5.4 ± 0.5	5.4 ± 0.4
Total cholesterol (mmol/L)	4.01 ± 0.44	4.36 ± 0.90		4.1 ± 0.3	4.0 ± 0.7
Fasting triglycerides (mmol/L)	1.10 ± 0.83	1.02 ± 0.55		1.2 ± 0.4	1.3 ± 0.5
HDL (mmol/L)	1.45 ± 0.47	1.46 ± 0.62		1.1 ± 0.2	1.1 ± 0.2
LDL (mmol/L)	2.19 ± 0.22	2.47 ± 0.84		2.6 ± 0.4	2.5 ± 0.7

**Table 2:** The top 20 lncRNAs with significant differential expression in white blood cells from both T1D patients and healthy controls.

Gene	C normalize	T1D normalize	Log_2_ fold change	FDR	Up/down	Gene name	Biotype	Position
ENSG00000273338	107.87	8.48	-3.67	7.91688*E* − 07	Down	RP11-386I14.4	Antisense	chr1:78004346-78004554:-
ENSG00000270069	433.51	76.07	-2.51	8.65909*E* − 06	Down	MIR222HG	lincRNA	chrX:45745211-45770274:-
ENSG00000269902	86.55	13.28	-2.70	0.000138695	Down	RP6-99M1.3	lincRNA	chrX:45764772-45765299:-
MSTRG.74858	80.41	9.89	-3.02	0.000569468	Down	—	linc	chr17:83203319-83204570:+
MSTRG.49388	15.38	0.32	-5.60	0.000600013	Down	—	linc	chr13:88709424-88744650:+
MSTRG.185495	1.27	18.66	3.88	0.000619542	Up	—	linc	chr9:128668273-128670326:-
ENSG00000267174	50.40	401.59	2.99	0.00066093	Up	CTC-510F12.4	3prime_overlapping	chr19:11300777-11324441:-
MSTRG.182419	91.26	17.30	-2.40	0.000702034	Down	—	linc	chr9:91193131-91198833:+
MSTRG.180334	0.62	13.17	4.41	0.000704429	Up	—	linc	chr9:61465105-61467945:+
MSTRG.71335	23.50	100.09	2.09	0.000976723	Up	—	linc	chr17:42195078-42198266:-
MSTRG.111791	28.91	116.47	2.01	0.000979217	Up	—	linc	chr22:41419090-41428731:+
MSTRG.125714	9.04	50.39	2.48	0.001001754	Up	—	Antisense	chr3:196215032-196228373:-
MSTRG.161229	28.27	137.55	2.28	0.001011298	Up	—	Antisense	chr7:27102269-27107589:+
MSTRG.73913	0.15	8.82	5.90	0.001038576	Up	—	Antisense	chr17:75346002-75347825:+
MSTRG.10587	4.81	30.79	2.68	0.001351147	Up	—	Antisense	chr1:156211503-156218677:-
MSTRG.147880	0.19	15.30	6.31	0.001402952	Up	—	linc	chr5:178650106-178668781:-
MSTRG.95087	97.19	17.17	-2.50	0.00144056	Down	—	linc	chr2:144660953-144665439:+
MSTRG.103146	618.92	214.32	-1.53	0.001589855	Down	—	Antisense	chr20:17570371-17583003:+
MSTRG.5412	50.35	311.44	2.63	0.001609361	Up	—	linc	chr1:65034964-65038215:-
MSTRG.80841	1.09	13.82	3.67	0.001677829	Up	—	linc	chr19:10959257-10960669:-

**Table 3:** Information of 12 lncRNAs validated with the expanded cohort in the present study and their potential orthologous sequences compared with mouse data.

lncRNA	Novel/known	Log_2_ value in the RNA-seq data	Significant difference in validation cohort	Orthologous sequences from mouse
ENSG00000269902	Known	-2.70	Yes	Not found
ENSG00000224515	Known	—	Yes	Not found
ENSG00000273338	Known	-3.67	No	Not found
ENSG00000267174	Known	2.99	Yes	Not found
MSTRG.128697	Novel	—	Yes	Not found
MSTRG.166799	Novel	1.11	Yes	chr6:39,016,934-39,045,231
MSTRG.128958	Novel	—	Yes	Not found
MSTRG.49388	Novel	-5.60	No	chr14:112,587,568-112,602,575
MSTRG.74858	Novel	-3.02	Yes	chr11:121,806,938-121,808,036
MSTRG.72098	Novel	3.71	Yes	chr11:95,398,836-95,397,976
MSTRG.23167	Novel	1.01	No	chr19:35,211,979-35,245,761
MSTRG.63013	Novel	2.55	Yes	chr17:23,644,824-23,660,240

**Table 4:** Four lncRNAs and their predicted mRNA targets identified.

lncRNAs	Predicted mRNAs
MSTRG.166799	PARP12
MSTRG.72098	SLC35B1, FAM117A, SPOP
ENSG00000267174	TMEM205, DOCK6, CCDC159, PLPPR2
MSTRG.63013	DNAJC21, KPNA3, SEPT2, THAP12, PSMD14, YME1L1, DHX29, RANBP6, CYCS, RECQL, BICDL2, THOC6, HCFC1R1, TNFRSF12A, IL32, CLDN9, G3BP2, FYTTD1, MRPL32, VPS26A, CGGBP1, IPO5, ZSCAN10, LA16c-380H5.3

## Data Availability

The datasets generated and analyzed in this study are not publicly available as written consent is required from the study participants, short of which sharing of individual-level phenotype data is prohibited.

## Supplementary Materials

Supplementary materials Figure S1: the conservation of novel lncRNAs and mRNAs among chromosomes. Figure S2: bioinformation analysis by enrichment analysis of pathways and GO terms for T1D-lncRNA using optimized data. The top 20 GO terms of lncRNAs enriched in (a) biological processes (BP), (b) molecular functions (MF), and (c) cellular components (CC). The number of enriched genes is indicated with size of the circle, the FDR ranges from red to green as expressed in different colors, and *p* value grows in the process of red to green calibration. Figure S3: bioinformation analysis by enrichment analysis of pathways and GO terms for T1D-mRNA using optimized data. The top 20 GO terms of mRNA enriched in (a) biological processes (BP), (b) molecular functions (MF), and (c) cellular components (CC). The number of enriched genes is indicated with size of the circle, the FDR ranges from red to green as expressed in different colors, and *p* value grows in the process of red to green calibration. Table S1: information of 393 significantly differentially expressed T1D-lncRNAs. The identified 393 differentially expressed T1D-lncRNAs (69 downregulated and 324 upregulated) between T1D and healthy control, in which 150 were antisense, 220 were intergenic (lincRNA), and 23 belonged to other subtypes (FDR: false discovery rate). Table S2: the information of 311 significantly differentially expressed T1D-mRNAs and healthy control. Table S3: the four positive lncRNAs and their targets of predicted mRNAs. Four lncRNAs were predicted with mRNA targets related to 32 genes. A total of 16 genes were included in the network of MSTRG.63013, with a correlation score of more than 0.9, and there were 8 genes within 50 kb of MSTRG.63013.
