# Supplementary material for: A Genome-Wide Analysis of Long Noncoding RNAs in Circulating Leukocytes and Their Differential Expression in Type 1 Diabetes Patients
Source: J Diabetes Res. 2020 Nov 25;2020:9010314. doi: 10.1155/2020/9010314 (PMC7710437; doi:10.1155/2020/9010314)
Supplement: Supplementary materials — Figure S1: the conservation of novel lncRNAs and mRNAs among chromosomes. Figure S2: bioinformation analysis by enrichment analysis of pathways and GO terms for T1D-lncRNA using optimized data. The top 20 GO terms of lncRNAs enriched in (a) biological processes (BP), (b) molecular functions (MF), and (c) cellular components (CC). The number of enriched genes is indicated with size of the circle, the FDR ranges from red to green as expressed in different colors, and p value grows in the process of red to green calibration. Figure S3: bioinformation analysis by enrichment analysis of pathways and GO terms for T1D-mRNA using optimized data. The top 20 GO terms of mRNA enriched in (a) biological processes (BP), (b) molecular functions (MF), and (c) cellular components (CC). The number of enriched genes is indicated with size of the circle, the FDR ranges from red to green as expressed in different colors, and p value grows in the process of red to green calibration. Table S1: information of 393 significantly differentially expressed T1D-lncRNAs. The identified 393 differentially expressed T1D-lncRNAs (69 downregulated and 324 upregulated) between T1D and healthy control, in which 150 were antisense, 220 were intergenic (lincRNA), and 23 belonged to other subtypes (FDR: false discovery rate). Table S2: the information of 311 significantly differentially expressed T1D-mRNAs and healthy control. Table S3: the four positive lncRNAs and their targets of predicted mRNAs. Four lncRNAs were predicted with mRNA targets related to 32 genes. A total of 16 genes were included in the network of MSTRG.63013, with a correlation score of more than 0.9, and there were 8 genes within 50 kb of MSTRG.63013. [file 9010314.f1.doc]

**Supplementary Figure legends**

FIGURE S1 The conservation of novel lncRNAs and mRNAs among chromosomes.

More than half of the novel lncRNA and mRNA transcripts have a conservation scores (CS) less than 0.1, which means absolute conservation in humans. Analysis of the distribution of transcripts on chromosomes demonstrates that novel lncRNAs and mRNAs are mainly distributed on chr1, chr2, chr3, chr4, chr5, and chr6 and also showed that most of lncRNAs and mRNAs that exhibited low CS in humans come from the same chromosome. (The ratio of transcript with CS ranges from 0 to 1.0. CS scores less than 0.1 are defined as low conserved sequence.)

**
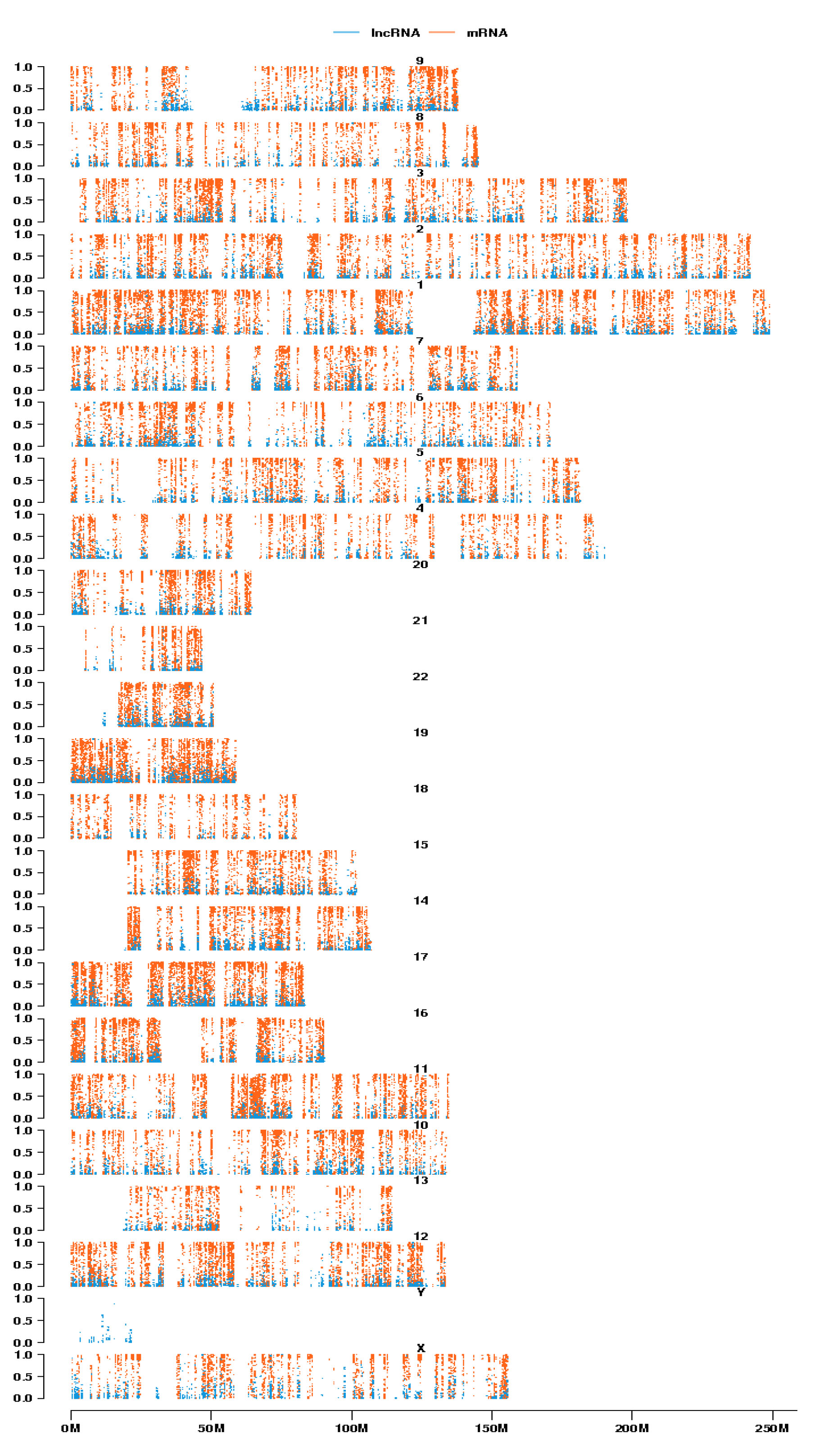
**

**FIGURE S2** Bioinformation analysis by enrichment analysis of pathways and GO terms for T1D-lncRNA using optimized data. The top 20 GO terms of lncRNAs enriched in a) biological processes (BP), b) molecular functions (MF), and c) cellular components (CC). The number of enriched genes is indicated with size of the circle, the FDR ranges from red to green as expressed in different colors, and p value grows in the process of red to green calibration.


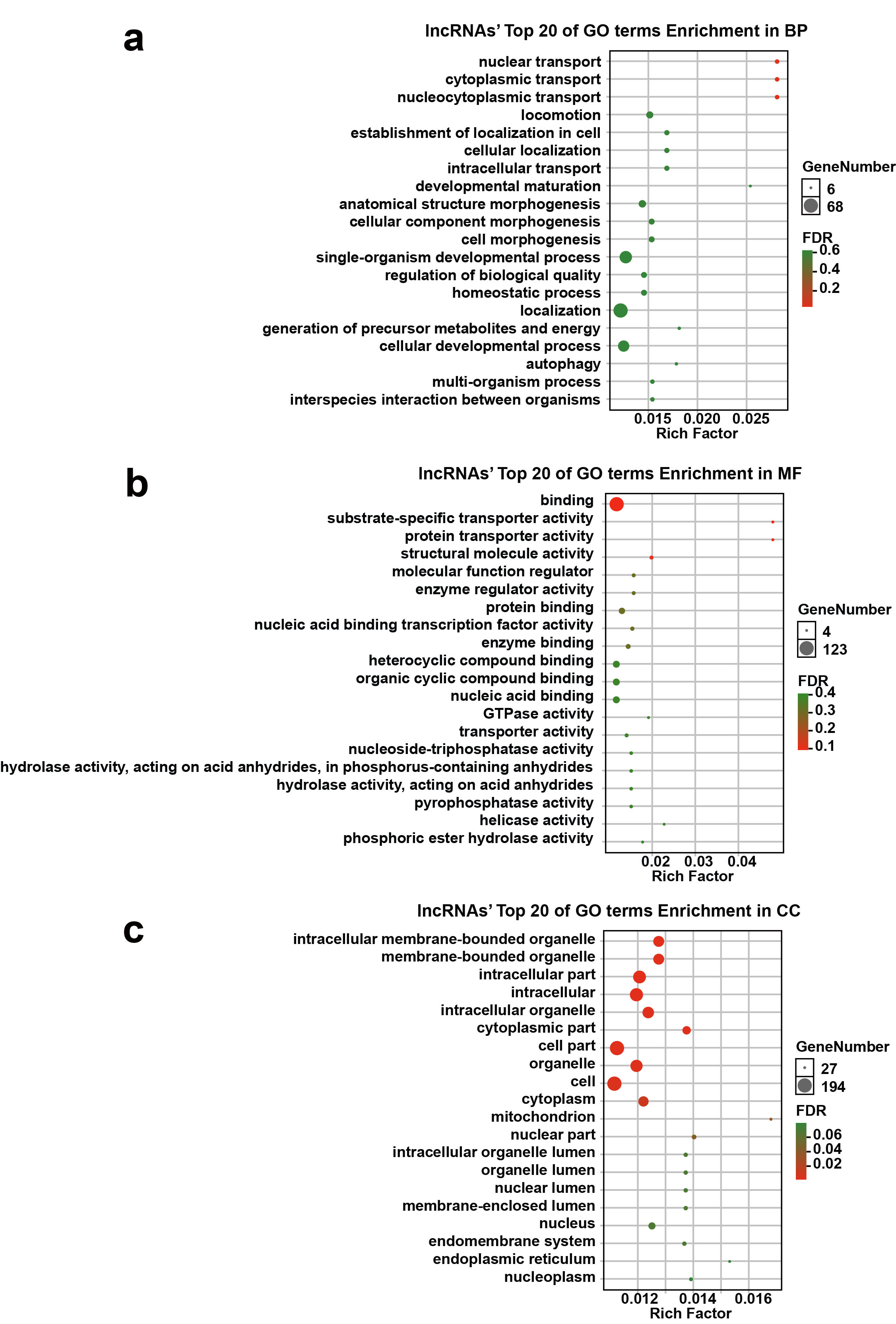


FIGURE S3 Bioinformation analysis by enrichment analysis of pathways and GO terms for T1D-mRNA using optimized data. The top 20 GO terms of mRNA enriched in a) biological processes (BP), b) molecular functions (MF), and c) cellular components (CC). The number of enriched genes is indicated with size of the circle, the FDR ranges from red to green as expressed in different colors, and p value grows in the process of red to green calibration.


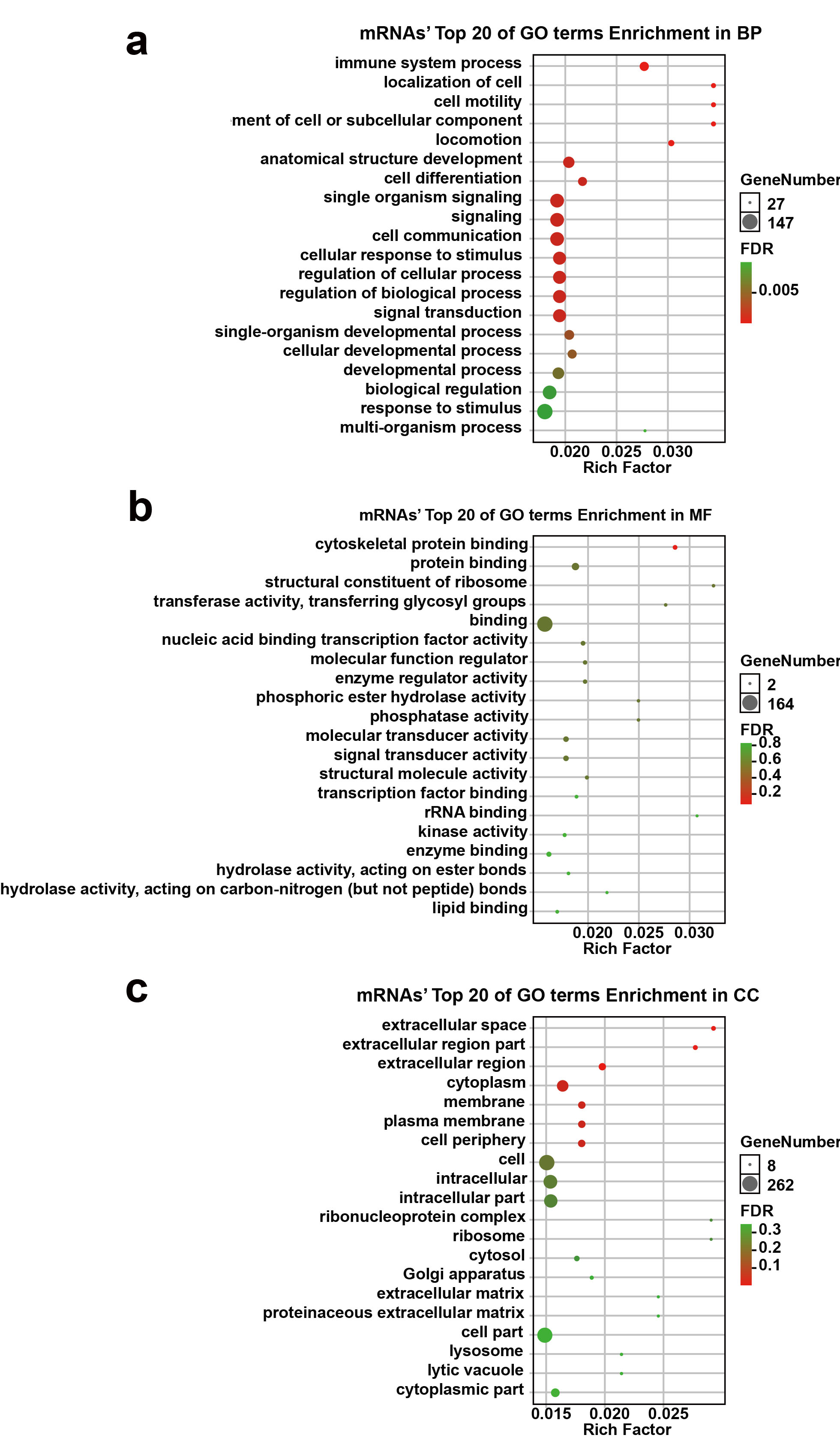


Supplemental Tables

Table S1 Information of 393 significantly differentially expressed T1D-lncRNAs

The identified 393 differentially expressed T1D-lncRNAs (69 down-regulated and 324 up-regulated) between T1D and healthy control, in which 150 were antisense, 220 were intergenic (lincRNA) and 23 belonged to other subtypes. (FDR: false discovery rate)

| **Gene** | **Biotype** | **Position** | **Control**  **normalize** | **T1D**  **normalize** | **Fold**  **Change** | ***p*-value** | **FDR** |
| --- | --- | --- | --- | --- | --- | --- | --- |
| ENSG00000273338 | antisense | chr1:78004346-78004554:- | 107.8691 | 8.479574 | 0.07861 | 1.97E-10 | 7.92E-07 |
| ENSG00000270069 | lincRNA | chrX:45745211-45770274:- | 433.5081 | 76.0749 | 0.175487 | 4.32E-09 | 8.66E-06 |
| ENSG00000269902 | lincRNA | chrX:45764772-45765299:- | 86.55289 | 13.28326 | 0.15347 | 1.04E-07 | 0.000139 |
| MSTRG.74858 | linc | chr17:83203319-83204570:+ | 80.41206 | 9.894132 | 0.123043 | 5.68E-07 | 0.000569 |
| MSTRG.49388 | linc | chr13:88709424-88744650:+ | 15.37565 | 0.317144 | 0.020626 | 1.05E-06 | 0.0006 |
| MSTRG.185495 | linc | chr9:128668273-128670326:- | 1.265087 | 18.66261 | 14.75203 | 7.72E-07 | 0.00062 |
| ENSG00000267174 | 3prime_overlapping_ncRNA | chr19:11300777-11324441:- | 50.39806 | 401.5903 | 7.968368 | 9.88E-07 | 0.000661 |
| MSTRG.182419 | linc | chr9:91193131-91198833:+ | 91.25823 | 17.30148 | 0.189588 | 1.40E-06 | 0.000702 |
| MSTRG.180334 | linc | chr9:61465105-61467945:+ | 0.619062 | 13.17377 | 21.28022 | 1.58E-06 | 0.000704 |
| MSTRG.71335 | linc | chr17:42195078-42198266:- | 23.50046 | 100.0947 | 4.259265 | 2.92E-06 | 0.000977 |
| MSTRG.111791 | linc | chr22:41419090-41428731:+ | 28.9085 | 116.4702 | 4.028926 | 3.17E-06 | 0.000979 |
| MSTRG.125714 | antisense | chr3:196215032-196228373:- | 9.037636 | 50.38675 | 5.575213 | 2.75E-06 | 0.001002 |
| MSTRG.161229 | antisense | chr7:27102269-27107589:+ | 28.27359 | 137.5454 | 4.8648 | 3.53E-06 | 0.001011 |
| MSTRG.73913 | antisense | chr17:75346002-75347825:+ | 0.1476 | 8.822027 | 59.76984 | 2.59E-06 | 0.001039 |
| MSTRG.10587 | antisense | chr1:156211503-156218677:- | 4.810828 | 30.79452 | 6.401085 | 5.05E-06 | 0.001351 |
| MSTRG.147880 | linc | chr5:178650106-178668781:- | 0.192849 | 15.30024 | 79.33797 | 5.94E-06 | 0.001403 |
| MSTRG.95087 | linc | chr2:144660953-144665439:+ | 97.18716 | 17.17463 | 0.176717 | 5.74E-06 | 0.001441 |
| MSTRG.103146 | antisense | chr20:17570371-17583003:+ | 618.923 | 214.323 | 0.346284 | 7.53E-06 | 0.00159 |
| MSTRG.5412 | linc | chr1:65034964-65038215:- | 50.34891 | 311.4423 | 6.185681 | 7.22E-06 | 0.001609 |
| MSTRG.80841 | linc | chr19:10959257-10960669:- | 1.087576 | 13.81944 | 12.70665 | 8.36E-06 | 0.001678 |
| MSTRG.126704 | linc | chr4:6886155-6888188:+ | 9.521184 | 59.00034 | 6.196744 | 1.09E-05 | 0.002091 |
| MSTRG.159327 | antisense | chr7:6405349-6450822:+ | 102.1373 | 464.9234 | 4.551944 | 1.22E-05 | 0.002127 |
| MSTRG.66083 | antisense | chr16:67998699-68001317:+ | 152.1987 | 722.5646 | 4.747507 | 1.28E-05 | 0.002132 |
| MSTRG.65777 | linc | chr16:56984544-56989411:- | 79.26222 | 266.6848 | 3.36459 | 1.18E-05 | 0.002146 |
| MSTRG.153609 | linc | chr6:89357830-89362898:- | 10.15942 | 49.4069 | 4.863161 | 1.37E-05 | 0.002203 |
| MSTRG.825 | antisense | chr1:9627245-9742715:+ | 1040.16 | 2717.601 | 2.612677 | 1.71E-05 | 0.002638 |
| MSTRG.54756 | linc | chr14:77503115-77505323:- | 2.71074 | 19.28048 | 7.112625 | 2.05E-05 | 0.003043 |
| MSTRG.72904 | linc | chr17:61935496-61937631:- | 0.980423 | 11.00725 | 11.22705 | 2.26E-05 | 0.003239 |
| MSTRG.83473 | antisense | chr19:41769850-41775741:- | 0.516711 | 9.016824 | 17.45042 | 2.47E-05 | 0.003422 |
| ENSG00000278330 | antisense | chr18:77112602-77115726:+ | 23.24199 | 2.821099 | 0.121379 | 2.64E-05 | 0.003535 |
| MSTRG.193308 | linc | chrX:118724910-118727398:- | 4.428524 | 26.60799 | 6.00832 | 2.91E-05 | 0.003761 |
| MSTRG.67209 | linc | chr16:85025712-85027615:- | 6.474033 | 31.44793 | 4.857549 | 3.47E-05 | 0.004347 |
| MSTRG.72098 | linc | chr17:49694654-49695686:+ | 1.659855 | 21.7155 | 13.08277 | 3.76E-05 | 0.004569 |
| ENSG00000276136 | lincRNA | chr12:32000375-32001222:+ | 64.39607 | 236.3406 | 3.670109 | 4.11E-05 | 0.004849 |
| MSTRG.20446 | linc | chr10:12270186-12271320:+ | 2.916909 | 20.25743 | 6.944827 | 4.31E-05 | 0.004939 |
| MSTRG.161948 | antisense | chr7:38010674-38071004:+ | 20.23844 | 2.995336 | 0.148002 | 5.27E-05 | 0.005562 |
| MSTRG.22946 | linc | chr10:89027822-89033111:+ | 60.99456 | 188.3493 | 3.08797 | 5.48E-05 | 0.005642 |
| MSTRG.17861 | linc | chr10:3785565-3787696:+ | 972.9775 | 293.544 | 0.301697 | 5.22E-05 | 0.005666 |
| MSTRG.189033 | antisense | chrX:44285879-44290261:- | 0.971957 | 11.14014 | 11.46155 | 5.17E-05 | 0.005759 |
| MSTRG.39530 | linc | chr12:56197580-56199781:- | 6.921968 | 34.41182 | 4.971393 | 5.84E-05 | 0.005858 |
| ENSG00000276216 | lincRNA | chr1:145281116-145281462:+ | 108.0692 | 25.88969 | 0.239566 | 6.47E-05 | 0.006043 |
| MSTRG.183247 | linc | chr9:105023405-105063275:- | 25.92618 | 185.5508 | 7.156892 | 6.19E-05 | 0.006057 |
| MSTRG.110430 | antisense | chr22:29730163-29730684:- | 0.492427 | 13.2475 | 26.90248 | 6.39E-05 | 0.006101 |
| MSTRG.22725 | linc | chr10:87486843-87489249:+ | 0.90237 | 9.387255 | 10.40289 | 7.01E-05 | 0.006116 |
| MSTRG.183248 | linc | chr9:105025478-105029529:- | 5.309566 | 39.95548 | 7.525187 | 6.96E-05 | 0.006209 |
| MSTRG.94162 | antisense | chr2:127445820-127461574:- | 8.847326 | 39.94352 | 4.514757 | 6.95E-05 | 0.006338 |
| MSTRG.67728 | linc | chr16:89968110-89969722:+ | 1.012457 | 9.781004 | 9.660661 | 7.44E-05 | 0.00635 |
| MSTRG.59262 | antisense | chr15:58446518-58451430:- | 3.731775 | 25.91734 | 6.945043 | 8.30E-05 | 0.006795 |
| MSTRG.80769 | linc | chr19:10347759-10348204:- | 0.774359 | 9.607998 | 12.40767 | 8.23E-05 | 0.006877 |
| MSTRG.31213 | antisense | chr11:73418913-73426723:+ | 228.313 | 615.4686 | 2.695724 | 9.14E-05 | 0.006918 |
| ENSG00000274737 | sense_intronic | chr12:47817451-47817966:- | 16.92569 | 61.50736 | 3.633965 | 9.06E-05 | 0.006991 |
| ENSG00000231485 | lincRNA | chr1:65066627-65067737:- | 5.494203 | 27.43731 | 4.993866 | 8.90E-05 | 0.007006 |
| MSTRG.128652 | linc | chr4:37877914-37888331:+ | 35.74348 | 171.069 | 4.78602 | 8.74E-05 | 0.007015 |
| MSTRG.13472 | linc | chr1:194220525-194230636:- | 283.0081 | 87.32232 | 0.308551 | 9.95E-05 | 0.007392 |
| MSTRG.116307 | antisense | chr3:48458614-48467681:- | 119.8212 | 316.1854 | 2.63881 | 0.000106 | 0.007739 |
| MSTRG.64433 | linc | chr16:28076685-28097390:- | 199.0141 | 883.9693 | 4.441741 | 0.000109 | 0.007781 |
| MSTRG.111881 | antisense | chr22:42120792-42125846:- | 1.904417 | 13.88709 | 7.292044 | 0.000111 | 0.007844 |
| MSTRG.21857 | linc | chr10:72328686-72366452:- | 33.82677 | 112.0883 | 3.313597 | 0.000116 | 0.007918 |
| MSTRG.80933 | antisense | chr19:11315420-11320164:- | 59.36294 | 349.8671 | 5.893695 | 0.000119 | 0.007937 |
| MSTRG.164855 | antisense | chr7:100156429-100158162:+ | 48.56553 | 144.584 | 2.977091 | 0.000116 | 0.008046 |
| MSTRG.81088 | antisense | chr19:13172244-13218650:+ | 23.73098 | 89.46969 | 3.770164 | 0.000135 | 0.00861 |
| MSTRG.182532 | linc | chr9:93044764-93049979:+ | 5.710508 | 39.15939 | 6.857427 | 0.000134 | 0.008668 |
| ENSG00000261367 | antisense | chr16:30107675-30110541:+ | 0.192849 | 7.902372 | 40.97703 | 0.000133 | 0.008763 |
| MSTRG.55835 | linc | chr14:98973454-99107682:- | 251.7534 | 631.9347 | 2.510134 | 0.000143 | 0.008963 |
| MSTRG.138541 | linc | chr5:39074483-39077107:+ | 38.86785 | 191.8299 | 4.935438 | 0.000154 | 0.00907 |
| MSTRG.180322 | linc | chr9:61893177-61894855:+ | 0.340449 | 7.210046 | 21.17806 | 0.000152 | 0.009096 |
| MSTRG.184438 | antisense | chr9:114322652-114333001:- | 388.9649 | 906.9943 | 2.331815 | 0.000148 | 0.009113 |
| MSTRG.179910 | antisense | chr9:40610962-40611807:+ | 7.822522 | 0.16091 | 0.02057 | 0.00015 | 0.009131 |
| MSTRG.141988 | antisense | chr5:95790546-95792781:- | 11.52331 | 42.23768 | 3.665414 | 0.000172 | 0.009988 |
| ENSG00000268729 | antisense | chr19:55312029-55312495:- | 0.331504 | 5.968261 | 18.00356 | 0.000181 | 0.010215 |
| MSTRG.63013 | linc | chr16:3037243-3046951:- | 45.92806 | 268.203 | 5.839633 | 0.000179 | 0.01028 |
| MSTRG.179846 | antisense | chr9:40059342-40106069:- | 1127.392 | 340.8429 | 0.302329 | 0.000186 | 0.010383 |
| ENSG00000232721 | lincRNA | chr1:143736066-143739506:+ | 0.1476 | 5.257319 | 35.6187 | 0.000194 | 0.01065 |
| MSTRG.35821 | antisense | chr12:1796345-1797664:+ | 21.84723 | 76.02969 | 3.48006 | 0.000209 | 0.010902 |
| MSTRG.53707 | linc | chr14:64674107-64703933:+ | 4.792399 | 35.65346 | 7.439584 | 0.000201 | 0.010923 |
| MSTRG.24156 | antisense | chr10:104036202-104039518:+ | 15.02378 | 56.12548 | 3.735776 | 0.000204 | 0.010939 |
| MSTRG.13786 | antisense | chr1:200345350-200347125:- | 2.054949 | 18.27008 | 8.89077 | 0.000209 | 0.011031 |
| ENSG00000130600 | processed_transcript | chr11:1995163-2001470:- | 13.30473 | 1.087037 | 0.081703 | 0.000216 | 0.011101 |
| ENSG00000270022 | lincRNA | chr22:42615244-42615907:+ | 86.20783 | 21.9522 | 0.254643 | 0.000223 | 0.011309 |
| MSTRG.110441 | linc | chr22:29779652-29781311:- | 0.499246 | 6.463916 | 12.94735 | 0.000243 | 0.012187 |
| ENSG00000272953 | lincRNA | chr7:5425770-5426401:+ | 4.890952 | 22.52328 | 4.605092 | 0.00026 | 0.012903 |
| MSTRG.148346 | linc | chr6:2798034-2799059:+ | 2.736685 | 15.69971 | 5.736759 | 0.000274 | 0.012931 |
| MSTRG.76257 | linc | chr18:21637627-21648684:- | 49.70965 | 195.0255 | 3.923291 | 0.000272 | 0.012976 |
| MSTRG.19754 | linc | chr10:29769847-29777336:- | 8.786761 | 0.600073 | 0.068293 | 0.000266 | 0.013021 |
| MSTRG.19495 | linc | chr10:27465297-27468217:+ | 0.672008 | 7.020484 | 10.44702 | 0.00027 | 0.013059 |
| MSTRG.83970 | antisense | chr19:46013762-46019049:- | 21.92567 | 69.4504 | 3.167539 | 0.000289 | 0.013477 |
| ENSG00000258820 | antisense | chr14:75259411-75271950:+ | 290.9377 | 70.11026 | 0.24098 | 0.000293 | 0.013517 |
| ENSG00000235728 | lincRNA | chr7:36781008-36782789:+ | 12.16093 | 1.240982 | 0.102047 | 0.000318 | 0.013575 |
| MSTRG.11371 | linc | chr1:161634393-161637895:- | 3.202972 | 72.35023 | 22.58847 | 0.000322 | 0.013586 |
| MSTRG.190993 | antisense | chrX:71349542-71352520:- | 13.63004 | 52.79481 | 3.873416 | 0.00031 | 0.013672 |
| MSTRG.73803 | linc | chr17:74487168-74517616:+ | 84.25128 | 249.8186 | 2.965161 | 0.000307 | 0.013675 |
| MSTRG.23568 | antisense | chr10:97761089-97766900:+ | 60.60105 | 163.0759 | 2.690974 | 0.000304 | 0.013699 |
| MSTRG.70558 | antisense | chr17:35790194-35793012:- | 5.489617 | 25.37852 | 4.623004 | 0.000318 | 0.013701 |
| MSTRG.54498 | linc | chr14:75298906-75303521:+ | 97.40563 | 23.43975 | 0.240641 | 0.000302 | 0.013777 |
| MSTRG.46819 | linc | chr13:37089912-37091146:- | 6.900395 | 48.42425 | 7.017606 | 0.000333 | 0.013783 |
| MSTRG.84405 | antisense | chr19:49551093-49565172:+ | 41.40262 | 123.4952 | 2.982787 | 0.000316 | 0.013801 |
| MSTRG.185500 | linc | chr9:128665373-128680368:- | 25.3549 | 82.2702 | 3.244746 | 0.000333 | 0.013903 |
| ENSG00000232912 | antisense | chr1:8424645-8434838:+ | 31.64724 | 97.48876 | 3.080482 | 0.000343 | 0.014053 |
| MSTRG.31440 | antisense | chr11:72759869-72814494:- | 653.2877 | 2431.186 | 3.721463 | 0.000354 | 0.014219 |
| MSTRG.166983 | linc | chr7:142841971-142855011:- | 1.815113 | 12.86177 | 7.085935 | 0.000352 | 0.014254 |
| ENSG00000264188 | sense_intronic | chr18:21661787-21662395:- | 3.979561 | 19.61574 | 4.929122 | 0.000374 | 0.014871 |
| MSTRG.30578 | antisense | chr11:65768007-65772614:- | 24.93132 | 155.9629 | 6.255704 | 0.000393 | 0.015028 |
| MSTRG.108059 | antisense | chr21:34517762-34538256:+ | 15.10989 | 55.04989 | 3.643302 | 0.000393 | 0.015146 |
| ENSG00000279727 | lincRNA | chr3:36819276-36822498:- | 107.0542 | 274.7623 | 2.566573 | 0.000401 | 0.015187 |
| MSTRG.746 | antisense | chr1:8951895-8971270:- | 342.2399 | 834.0451 | 2.437019 | 0.000391 | 0.015249 |
| MSTRG.166801 | antisense | chr7:140012852-140018111:- | 13.31448 | 52.79283 | 3.965069 | 0.000388 | 0.01527 |
| MSTRG.21851 | linc | chr10:72237497-72241833:+ | 33.89711 | 97.78647 | 2.884802 | 0.000414 | 0.01551 |
| MSTRG.72756 | linc | chr17:59408838-59433609:+ | 28.80372 | 86.36205 | 2.998295 | 0.00046 | 0.01677 |
| MSTRG.54053 | antisense | chr14:70313830-70342922:+ | 11.03801 | 0.940805 | 0.085233 | 0.000454 | 0.016869 |
| MSTRG.39819 | antisense | chr12:57466810-57471270:- | 105.6774 | 441.537 | 4.178157 | 0.00046 | 0.016918 |
| MSTRG.99519 | linc | chr2:207807527-207812837:- | 3.856044 | 29.10609 | 7.548174 | 0.00048 | 0.017029 |
| ENSG00000265791 | sense_intronic | chr17:30781493-30782221:- | 8.468879 | 32.96 | 3.891896 | 0.000473 | 0.017085 |
| MSTRG.167920 | linc | chr7:153735422-153744970:+ | 9.395968 | 0.785014 | 0.083548 | 0.000479 | 0.01717 |
| MSTRG.102592 | linc | chr20:5504620-5506455:- | 2.353099 | 13.08149 | 5.559259 | 0.00049 | 0.017256 |
| MSTRG.38036 | linc | chr12:31995136-32037418:+ | 503.5815 | 1854.223 | 3.682071 | 0.000508 | 0.017425 |
| MSTRG.135829 | linc | chr4:183339333-183342777:- | 13.02357 | 1.394932 | 0.107108 | 0.0005 | 0.017446 |
| MSTRG.110125 | linc | chr22:24531136-24539457:+ | 8.190745 | 37.69272 | 4.601866 | 0.000507 | 0.017539 |
| MSTRG.180437 | linc | chr9:62798833-62801387:+ | 208.7924 | 469.046 | 2.24647 | 0.00052 | 0.017697 |
| MSTRG.121783 | antisense | chr3:136862215-136862710:- | 1.146092 | 8.961306 | 7.819009 | 0.000527 | 0.017769 |
| MSTRG.69585 | linc | chr17:19898607-19901969:- | 164.1005 | 372.9721 | 2.272827 | 0.000532 | 0.017801 |
| ENSG00000261218 | lincRNA | chr16:81738248-81767868:+ | 32.16348 | 93.68611 | 2.91281 | 0.000542 | 0.017977 |
| MSTRG.145481 | linc | chr5:139630842-139641245:+ | 192.6881 | 454.9393 | 2.361014 | 0.000555 | 0.01812 |
| MSTRG.119201 | linc | chr3:105280540-105287680:- | 17.873 | 60.54249 | 3.387371 | 0.000551 | 0.018124 |
| MSTRG.63104 | linc | chr16:4540405-4541587:+ | 0.1476 | 4.331679 | 29.34742 | 0.000573 | 0.018551 |
| ENSG00000279199 | TEC | chr17:40113215-40115442:- | 2.319432 | 12.69126 | 5.471711 | 0.000578 | 0.018554 |
| MSTRG.11326 | linc | chr1:161972044-161977537:+ | 18.70232 | 57.38648 | 3.068415 | 0.000586 | 0.018678 |
| MSTRG.47717 | linc | chr13:49639058-49671747:+ | 187.5157 | 649.2035 | 3.46213 | 0.000603 | 0.019056 |
| MSTRG.62902 | antisense | chr16:2785942-2791742:+ | 0.656561 | 7.139963 | 10.87479 | 0.000625 | 0.01945 |
| MSTRG.103519 | linc | chr20:23085665-23095514:+ | 3.941118 | 17.66971 | 4.483426 | 0.000638 | 0.019536 |
| ENSG00000248773 | sense_intronic | chr3:140972744-140973255:+ | 49.35247 | 14.49551 | 0.293714 | 0.000624 | 0.019559 |
| ENSG00000224116 | antisense | chr7:41693916-41779388:+ | 12.6429 | 1.828729 | 0.144645 | 0.000638 | 0.019681 |
| MSTRG.93076 | linc | chr2:111187202-111187998:+ | 0.340449 | 5.467163 | 16.05869 | 0.000696 | 0.021172 |
| MSTRG.104409 | antisense | chr20:35635089-35646991:- | 362.6763 | 785.9712 | 2.167143 | 0.000702 | 0.021189 |
| ENSG00000274902 | lincRNA | chr12:47731908-47732351:+ | 6.186165 | 25.36573 | 4.100397 | 0.000714 | 0.021371 |
| ENSG00000264007 | sense_intronic | chr17:29621617-29622254:- | 2.233887 | 12.78932 | 5.725143 | 0.000741 | 0.021398 |
| MSTRG.180491 | linc | chr9:63814492-63817650:- | 31.44104 | 88.05304 | 2.800576 | 0.000725 | 0.021399 |
| MSTRG.15600 | linc | chr1:226042936-226059382:- | 29.38727 | 84.16933 | 2.864143 | 0.000738 | 0.021459 |
| MSTRG.183281 | antisense | chr9:105707806-105713053:+ | 346.4714 | 149.9251 | 0.43272 | 0.000722 | 0.021474 |
| MSTRG.93732 | linc | chr2:120223519-120232968:- | 155.4525 | 485.3374 | 3.122095 | 0.000734 | 0.021502 |
| MSTRG.84892 | linc | chr19:54554302-54565981:- | 0.168565 | 4.296642 | 25.48957 | 0.000753 | 0.021584 |
| MSTRG.59140 | antisense | chr15:55169618-55354124:- | 171.1346 | 396.8134 | 2.318721 | 0.000831 | 0.021659 |
| ENSG00000279444 | TEC | chr12:63151065-63154006:+ | 10.2261 | 0.878374 | 0.085895 | 0.000839 | 0.021712 |
| MSTRG.116691 | antisense | chr3:52218973-52226065:+ | 13.8377 | 45.91681 | 3.318239 | 0.000823 | 0.02173 |
| ENSG00000280181 | TEC | chr12:64709458-64710513:+ | 15.59694 | 62.94545 | 4.035755 | 0.000831 | 0.021784 |
| ENSG00000257433 | antisense | chr12:47706085-47742294:+ | 52.64681 | 176.6592 | 3.355554 | 0.000793 | 0.021802 |
| ENSG00000272506 | lincRNA | chr1:65003470-65004087:- | 11.54896 | 40.69998 | 3.524125 | 0.000783 | 0.021811 |
| MSTRG.90359 | linc | chr2:65272261-65285448:+ | 470.876 | 1063.623 | 2.258819 | 0.000849 | 0.02183 |
| MSTRG.23644 | antisense | chr10:97346175-97435046:- | 11.37594 | 54.02062 | 4.748675 | 0.000822 | 0.02184 |
| ENSG00000270640 | sense_intronic | chr2:28396815-28397110:+ | 165.0977 | 50.38488 | 0.305182 | 0.000861 | 0.021876 |
| MSTRG.159431 | linc | chr7:6103517-6104660:- | 10.45948 | 38.03684 | 3.636591 | 0.000792 | 0.021923 |
| MSTRG.81195 | linc | chr19:12311243-12313735:- | 3.391584 | 18.29 | 5.392759 | 0.00082 | 0.02193 |
| MSTRG.108623 | linc | chr21:42784049-42792158:+ | 5.567459 | 37.97442 | 6.820782 | 0.000803 | 0.021932 |
| ENSG00000270055 | sense_intronic | chr15:30487963-30490313:+ | 508.0469 | 235.5349 | 0.463609 | 0.000858 | 0.021933 |
| MSTRG.170267 | antisense | chr8:28770038-28771987:+ | 21.83625 | 68.87604 | 3.154207 | 0.000782 | 0.021951 |
| ENSG00000265206 | antisense | chr17:58330884-58332508:- | 285.5067 | 716.6961 | 2.51026 | 0.00081 | 0.021973 |
| ENSG00000251022 | antisense | chr4:82893009-82900960:- | 986.2226 | 452.8299 | 0.459156 | 0.000876 | 0.02198 |
| MSTRG.71753 | linc | chr17:45490885-45491658:+ | 7.747009 | 30.49015 | 3.935731 | 0.00078 | 0.022044 |
| ENSG00000267512 | antisense | chr19:13139617-13141147:- | 0.939976 | 7.464243 | 7.940887 | 0.000819 | 0.02205 |
| MSTRG.98106 | antisense | chr2:190694763-190700937:+ | 30.80667 | 122.2482 | 3.968237 | 0.000875 | 0.022088 |
| MSTRG.167609 | antisense | chr7:150450476-150451324:- | 4.705098 | 19.34814 | 4.112165 | 0.000779 | 0.022158 |
| ENSG00000233096 | lincRNA | chr3:40970541-40971578:- | 5.597781 | 0.155791 | 0.027831 | 0.000901 | 0.022468 |
| MSTRG.12636 | linc | chr1:173926307-173930515:- | 66.3445 | 164.4134 | 2.478176 | 0.000919 | 0.022754 |
| MSTRG.13433 | linc | chr1:193869629-193952348:+ | 165.4283 | 55.9345 | 0.338119 | 0.000934 | 0.023005 |
| MSTRG.38251 | antisense | chr12:40191124-40210961:- | 55.68252 | 272.148 | 4.887495 | 0.000948 | 0.023209 |
| MSTRG.11365 | antisense | chr1:161512561-161525557:- | 3.320718 | 31.69176 | 9.543649 | 0.000958 | 0.023289 |
| ENSG00000224307 | lincRNA | chr9:129282458-129285728:+ | 37.60123 | 104.9154 | 2.790213 | 0.000982 | 0.023741 |
| ENSG00000259687 | lincRNA | chr14:75294404-75296638:+ | 130.1084 | 41.90566 | 0.322083 | 0.00103 | 0.024036 |
| MSTRG.194211 | linc | chrX:123943092-123945902:- | 0.479159 | 6.396539 | 13.3495 | 0.001043 | 0.02406 |
| MSTRG.138968 | linc | chr5:50174899-50206288:- | 736.0519 | 313.9728 | 0.426563 | 0.001003 | 0.024097 |
| ENSG00000230107 | lincRNA | chr22:42438023-42446195:+ | 2.377661 | 11.86995 | 4.992279 | 0.001029 | 0.024139 |
| MSTRG.2440 | linc | chr1:30101880-30130717:- | 20.76321 | 148.9484 | 7.173668 | 0.001042 | 0.024179 |
| MSTRG.39220 | linc | chr12:50758567-50759169:+ | 0.643346 | 7.07505 | 10.99727 | 0.001014 | 0.024217 |
| MSTRG.55786 | linc | chr14:98048478-98049892:- | 2.303162 | 12.51922 | 5.435665 | 0.001021 | 0.024238 |
| MSTRG.98806 | antisense | chr2:197557349-197572416:+ | 76.82079 | 183.594 | 2.3899 | 0.001028 | 0.024272 |
| MSTRG.1250 | antisense | chr1:15825336-15832696:- | 21.60808 | 80.07279 | 3.705687 | 0.001065 | 0.024414 |
| MSTRG.30000 | linc | chr11:47452518-47462678:- | 73.86282 | 184.0317 | 2.491533 | 0.001072 | 0.024444 |
| MSTRG.111888 | linc | chr22:42446987-42450777:+ | 3.262985 | 14.89686 | 4.56541 | 0.001103 | 0.025009 |
| MSTRG.49721 | linc | chr13:99395841-99398541:- | 4.571087 | 18.22448 | 3.986903 | 0.001146 | 0.025833 |
| MSTRG.89063 | linc | chr2:43135241-43145678:- | 26.29115 | 77.24043 | 2.937887 | 0.001164 | 0.026094 |
| MSTRG.82703 | linc | chr19:35455075-35456852:+ | 2.35008 | 28.24762 | 12.01985 | 0.001178 | 0.026126 |
| MSTRG.77486 | antisense | chr18:45743383-45747400:+ | 39.50765 | 11.86626 | 0.300354 | 0.001176 | 0.026211 |
| ENSG00000253430 | sense_intronic | chr8:26422593-26423929:+ | 1.882024 | 10.78284 | 5.729384 | 0.001208 | 0.026639 |
| MSTRG.1963 | linc | chr1:21210733-21217072:- | 28.77437 | 113.3905 | 3.940676 | 0.001227 | 0.02677 |
| MSTRG.38028 | linc | chr12:31865838-31870589:- | 18.38565 | 56.25566 | 3.059759 | 0.001237 | 0.026834 |
| MSTRG.59674 | antisense | chr15:65895307-65897459:+ | 59.74942 | 147.3483 | 2.466104 | 0.001227 | 0.026914 |
| MSTRG.181523 | antisense | chr9:78695042-78853673:+ | 1.340409 | 9.864538 | 7.35935 | 0.001257 | 0.026974 |
| ENSG00000231826 | lincRNA | chr2:43027853-43039547:- | 4.957495 | 21.02583 | 4.24122 | 0.001294 | 0.027036 |
| ENSG00000271840 | lincRNA | chr1:22100613-22101360:+ | 47.89014 | 135.9008 | 2.837761 | 0.001255 | 0.02708 |
| ENSG00000275371 | antisense | chr16:30110895-30111955:+ | 3.467964 | 21.91579 | 6.319496 | 0.001269 | 0.027096 |
| MSTRG.17812 | linc | chr10:3467535-3469984:+ | 6.121765 | 28.81234 | 4.706541 | 0.001292 | 0.027149 |
| MSTRG.47689 | antisense | chr13:48480787-48485893:- | 52.00565 | 126.2939 | 2.428465 | 0.001286 | 0.027156 |
| MSTRG.151472 | antisense | chr6:41146287-41178223:+ | 421.7758 | 168.5743 | 0.399678 | 0.00128 | 0.027178 |
| MSTRG.543 | antisense | chr1:8000636-8004482:+ | 7.941407 | 1.058045 | 0.133231 | 0.001334 | 0.027305 |
| MSTRG.117056 | linc | chr3:57937488-57946101:+ | 24.28165 | 67.31113 | 2.772099 | 0.001313 | 0.02731 |
| ENSG00000260019 | lincRNA | chr17:27929548-27974223:- | 7.893983 | 0.893044 | 0.11313 | 0.001329 | 0.027352 |
| MSTRG.74632 | antisense | chr17:81552455-81555280:+ | 30.18266 | 81.1348 | 2.688126 | 0.001327 | 0.027456 |
| MSTRG.36176 | antisense | chr12:6989528-6995257:+ | 4.476114 | 17.07038 | 3.813661 | 0.001366 | 0.027821 |
| MSTRG.10585 | linc | chr1:156180278-156191023:+ | 184.9124 | 605.7183 | 3.275703 | 0.001382 | 0.028008 |
| MSTRG.72535 | antisense | chr17:57909239-57933850:- | 78.88823 | 256.3727 | 3.249822 | 0.001417 | 0.028566 |
| ENSG00000186594 | lincRNA | chr17:1711493-1717174:- | 796.8704 | 321.4281 | 0.403363 | 0.001456 | 0.029218 |
| MSTRG.68116 | linc | chr17:1629558-1629982:- | 0.799195 | 10.22381 | 12.79263 | 0.001467 | 0.029279 |
| MSTRG.60140 | linc | chr15:69971436-69975793:- | 1.905043 | 12.29374 | 6.453261 | 0.001481 | 0.029416 |
| MSTRG.96089 | linc | chr2:159307052-159312559:- | 14.168 | 55.09075 | 3.888394 | 0.001577 | 0.02944 |
| MSTRG.89605 | antisense | chr2:54957708-54961159:- | 11.37679 | 48.65917 | 4.277055 | 0.001571 | 0.029451 |
| MSTRG.64351 | linc | chr16:28331011-28334012:+ | 4.254714 | 17.93648 | 4.215672 | 0.00149 | 0.029462 |
| ENSG00000280003 | TEC | chr3:52217016-52219012:- | 11.85431 | 48.69093 | 4.107446 | 0.001601 | 0.029477 |
| MSTRG.38692 | antisense | chr12:47742772-47747243:+ | 24.29284 | 73.63267 | 3.031044 | 0.001596 | 0.029516 |
| MSTRG.60283 | antisense | chr15:64506449-64592774:+ | 0.625881 | 6.138187 | 9.807268 | 0.00159 | 0.029535 |
| MSTRG.149960 | linc | chr6:26486395-26487739:- | 0.1476 | 3.544931 | 24.01715 | 0.001561 | 0.029557 |
| ENSG00000225195 | sense_intronic | chr12:64628344-64629976:+ | 24.52164 | 67.91441 | 2.769571 | 0.001569 | 0.029559 |
| MSTRG.13431 | antisense | chr1:193756234-193757080:+ | 26.47573 | 6.609124 | 0.24963 | 0.001559 | 0.029659 |
| MSTRG.65145 | linc | chr16:50658948-50663258:- | 60.068 | 221.7343 | 3.691389 | 0.001555 | 0.029719 |
| ENSG00000245105 | antisense | chr12:9065177-9068060:+ | 720.7665 | 343.3141 | 0.476318 | 0.001529 | 0.029788 |
| ENSG00000276241 | antisense | chr17:36116177-36177510:+ | 42.21706 | 11.26052 | 0.266729 | 0.001553 | 0.029821 |
| MSTRG.21653 | antisense | chr10:63161563-63182887:+ | 11.19931 | 38.2107 | 3.411879 | 0.001525 | 0.029853 |
| MSTRG.55369 | antisense | chr14:92691925-92696155:+ | 307.396 | 664.849 | 2.162842 | 0.001548 | 0.029863 |
| MSTRG.120650 | linc | chr3:122730938-122733204:+ | 9.166753 | 31.87554 | 3.477299 | 0.001542 | 0.029903 |
| ENSG00000279463 | sense_intronic | chr13:73844683-73845130:- | 123.563 | 48.56498 | 0.393038 | 0.001632 | 0.029913 |
| MSTRG.3691 | antisense | chr1:45399609-45419514:- | 86.97789 | 199.1272 | 2.2894 | 0.001521 | 0.029928 |
| ENSG00000269246 | lincRNA | chr19:39341773-39341945:- | 7.521937 | 30.8237 | 4.097841 | 0.001683 | 0.03069 |
| MSTRG.22658 | linc | chr10:86537923-86540149:- | 0.967101 | 6.511762 | 6.733283 | 0.001715 | 0.030997 |
| MSTRG.2659 | antisense | chr1:32860908-32864438:+ | 19.19509 | 55.20849 | 2.876177 | 0.001711 | 0.031069 |
| MSTRG.22726 | linc | chr10:87496228-87496570:+ | 0.137833 | 3.850373 | 27.93515 | 0.001737 | 0.031263 |
| MSTRG.84398 | linc | chr19:49549673-49554377:- | 9.146792 | 39.41827 | 4.309518 | 0.001788 | 0.031606 |
| MSTRG.812 | linc | chr1:9910602-9911516:+ | 1.567378 | 10.22668 | 6.524704 | 0.001772 | 0.031608 |
| ENSG00000236242 | antisense | chr13:109163902-109201483:- | 4.278338 | 18.45739 | 4.31415 | 0.001764 | 0.03161 |
| MSTRG.61205 | antisense | chr15:81315901-81323301:+ | 305.1434 | 709.0142 | 2.323544 | 0.001782 | 0.031647 |
| MSTRG.106237 | antisense | chr20:60081563-60087668:+ | 26.60532 | 71.28281 | 2.679269 | 0.001846 | 0.03166 |
| MSTRG.1260 | antisense | chr1:15941749-15943334:+ | 17.23942 | 51.21584 | 2.970856 | 0.001807 | 0.031672 |
| ENSG00000204625 | lincRNA | chr6:29975112-29978410:+ | 2.248621 | 13.67353 | 6.080853 | 0.001802 | 0.031722 |
| ENSG00000269892 | lincRNA | chr12:6742985-6743641:+ | 6.520665 | 23.04667 | 3.534404 | 0.001845 | 0.031782 |
| MSTRG.556 | linc | chr1:7863299-7913280:- | 216.7692 | 736.3339 | 3.396857 | 0.001871 | 0.031811 |
| MSTRG.2188 | linc | chr1:27044913-27064389:+ | 0.16294 | 3.657719 | 22.4483 | 0.001863 | 0.031813 |
| MSTRG.56358 | antisense | chr14:102368357-102394888:- | 17.97533 | 87.18696 | 4.850367 | 0.001951 | 0.031834 |
| MSTRG.139451 | linc | chr5:57169734-57173724:- | 122.226 | 46.94273 | 0.384065 | 0.001843 | 0.031884 |
| MSTRG.149669 | antisense | chr6:20400376-20402498:- | 17.61199 | 3.414838 | 0.193893 | 0.001837 | 0.031905 |
| MSTRG.61698 | antisense | chr15:89821456-89836802:+ | 10.01294 | 51.29017 | 5.122388 | 0.001949 | 0.031924 |
| MSTRG.123931 | linc | chr3:172867586-172871511:+ | 0.983795 | 7.075469 | 7.192016 | 0.001936 | 0.031966 |
| MSTRG.9012 | linc | chr1:114380013-114383006:- | 0.73593 | 6.710737 | 9.118719 | 0.001833 | 0.031979 |
| MSTRG.82541 | linc | chr19:34242693-34244086:+ | 0.817483 | 6.726943 | 8.228846 | 0.001931 | 0.032019 |
| MSTRG.141026 | linc | chr5:81818225-81845739:+ | 4.096975 | 31.8208 | 7.766902 | 0.001947 | 0.032022 |
| ENSG00000279693 | TEC | chr16:68367325-68370262:- | 7.658008 | 27.28923 | 3.56349 | 0.001911 | 0.032085 |
| MSTRG.125728 | linc | chr3:196443949-196458686:- | 16.83287 | 48.23859 | 2.865738 | 0.001928 | 0.032101 |
| MSTRG.31454 | linc | chr11:72793790-72810827:+ | 10.71246 | 33.33703 | 3.111985 | 0.001977 | 0.032116 |
| MSTRG.16103 | linc | chr1:230552207-230556500:- | 3.877544 | 16.80573 | 4.334116 | 0.001906 | 0.032135 |
| ENSG00000273139 | lincRNA | chr22:20320739-20321203:+ | 2.735132 | 13.19692 | 4.824967 | 0.001925 | 0.032185 |
| MSTRG.30136 | linc | chr11:62025111-62029411:+ | 274.6499 | 92.48794 | 0.336748 | 0.001903 | 0.032223 |
| MSTRG.116382 | linc | chr3:49355335-49356922:- | 318.9644 | 123.6568 | 0.387682 | 0.002008 | 0.032494 |
| MSTRG.116694 | linc | chr3:52251062-52260792:+ | 39.35877 | 114.8921 | 2.919097 | 0.002037 | 0.032824 |
| MSTRG.184314 | antisense | chr9:112483558-112484577:+ | 2.289329 | 11.32942 | 4.948797 | 0.002053 | 0.032831 |
| MSTRG.142970 | linc | chr5:109869957-109870453:+ | 3.742693 | 14.67721 | 3.921563 | 0.00205 | 0.032912 |
| MSTRG.183250 | linc | chr9:105045268-105049958:- | 10.97976 | 85.76892 | 7.811549 | 0.002069 | 0.032956 |
| MSTRG.22781 | antisense | chr10:87864018-87864678:- | 3.213523 | 13.99892 | 4.356252 | 0.002099 | 0.033291 |
| MSTRG.16534 | linc | chr1:235654423-235660031:- | 59.54665 | 173.4967 | 2.913626 | 0.002136 | 0.033347 |
| MSTRG.133801 | antisense | chr4:139033397-139042812:- | 110.3162 | 331.2376 | 3.002619 | 0.002122 | 0.033388 |
| ENSG00000261043 | lincRNA | chr15:75759501-75762405:- | 1.153363 | 20.12594 | 17.44978 | 0.002117 | 0.03345 |
| MSTRG.64597 | linc | chr16:30804907-30809600:+ | 10.35861 | 33.45727 | 3.229899 | 0.002135 | 0.033461 |
| MSTRG.77770 | antisense | chr18:49816683-49821978:+ | 30.16758 | 78.2032 | 2.592292 | 0.002162 | 0.033502 |
| ENSG00000273599 | antisense | chr10:124996064-125001491:+ | 25.66207 | 71.14177 | 2.772254 | 0.002157 | 0.033549 |
| MSTRG.101510 | linc | chr2:236537151-236550446:+ | 78.87559 | 195.1385 | 2.474004 | 0.002188 | 0.033641 |
| MSTRG.40287 | antisense | chr12:64699215-64741058:+ | 340.839 | 1253.458 | 3.677566 | 0.002199 | 0.033686 |
| MSTRG.127367 | antisense | chr4:10143081-10146587:+ | 3.7015 | 15.61055 | 4.217359 | 0.002187 | 0.033755 |
| MSTRG.179264 | linc | chr9:33374436-33376085:+ | 2.160622 | 9.699765 | 4.48934 | 0.002238 | 0.034022 |
| MSTRG.163167 | linc | chr7:66964205-66970222:+ | 1.87383 | 11.58842 | 6.18435 | 0.00223 | 0.034028 |
| MSTRG.110365 | antisense | chr22:29100431-29111356:+ | 62.73028 | 273.883 | 4.366042 | 0.002298 | 0.034793 |
| ENSG00000233214 | lincRNA | chr19:35424062-35424652:- | 4.07963 | 19.04039 | 4.667187 | 0.002328 | 0.035123 |
| ENSG00000271327 | lincRNA | chr12:89367807-89369301:+ | 5.017675 | 18.71543 | 3.7299 | 0.002359 | 0.035317 |
| MSTRG.166966 | linc | chr7:140924883-140927109:+ | 92.32876 | 20.03804 | 0.217029 | 0.002352 | 0.035354 |
| ENSG00000257809 | antisense | chr12:56150796-56158220:- | 3.245536 | 13.90221 | 4.283486 | 0.002469 | 0.035388 |
| MSTRG.81368 | antisense | chr19:16135156-16138551:+ | 30.83925 | 78.27804 | 2.53826 | 0.002465 | 0.035452 |
| ENSG00000226640 | lincRNA | chr1:193678894-193727035:+ | 16.57512 | 3.956538 | 0.238703 | 0.002457 | 0.035474 |
| ENSG00000236772 | antisense | chr20:32449755-32453607:+ | 1.44549 | 8.271598 | 5.722351 | 0.002489 | 0.035544 |
| ENSG00000233875 | antisense | chr1:154579065-154579663:- | 18.05499 | 51.95037 | 2.877341 | 0.002447 | 0.035579 |
| ENSG00000260859 | sense_intronic | chr16:84828263-84829242:+ | 3.919819 | 18.37702 | 4.688233 | 0.00243 | 0.035583 |
| MSTRG.111972 | antisense | chr22:43109567-43111354:- | 1.033422 | 7.064375 | 6.835907 | 0.002456 | 0.035586 |
| MSTRG.187021 | antisense | chrX:10018875-10037317:+ | 26.89354 | 120.3335 | 4.47444 | 0.002443 | 0.035657 |
| MSTRG.170575 | linc | chr8:38061085-38062605:+ | 3.826102 | 22.36187 | 5.844557 | 0.002429 | 0.035705 |
| MSTRG.24060 | linc | chr10:102816872-102826091:+ | 13.93127 | 45.42677 | 3.260778 | 0.002424 | 0.035758 |
| MSTRG.149102 | antisense | chr6:6687164-6706769:+ | 7.975302 | 35.20634 | 4.414421 | 0.002514 | 0.03577 |
| MSTRG.90771 | antisense | chr2:70553217-70569779:+ | 0.155297 | 3.652689 | 23.52062 | 0.002423 | 0.035875 |
| MSTRG.109789 | antisense | chr22:23146395-23153690:- | 20.97772 | 93.36427 | 4.450639 | 0.00253 | 0.035875 |
| MSTRG.154388 | antisense | chr6:106297287-106304946:- | 599.241 | 295.6813 | 0.493426 | 0.00254 | 0.035889 |
| MSTRG.183249 | linc | chr9:105036316-105061582:- | 22.37168 | 147.5482 | 6.595313 | 0.002422 | 0.035999 |
| MSTRG.73651 | antisense | chr17:73195686-73201020:- | 78.82603 | 170.918 | 2.168293 | 0.002414 | 0.036013 |
| MSTRG.150427 | linc | chr6:31563901-31566008:- | 10.13872 | 31.25434 | 3.082672 | 0.002579 | 0.036186 |
| ENSG00000235908 | sense_intronic | chr3:49365145-49367006:- | 13.89419 | 40.21057 | 2.894056 | 0.002578 | 0.036301 |
| MSTRG.128957 | linc | chr4:40409396-40416959:- | 24.16137 | 68.44062 | 2.832646 | 0.00265 | 0.03705 |
| MSTRG.184237 | linc | chr9:112193628-112195696:- | 5.24828 | 19.67837 | 3.74949 | 0.002696 | 0.037435 |
| MSTRG.116308 | linc | chr3:48513740-48516990:- | 30.35968 | 127.879 | 4.212132 | 0.002688 | 0.037459 |
| MSTRG.165064 | antisense | chr7:103328437-103334335:- | 208.7944 | 93.18704 | 0.44631 | 0.002759 | 0.037921 |
| MSTRG.121315 | linc | chr3:129080251-129082986:- | 0.355789 | 4.58497 | 12.88678 | 0.002772 | 0.03796 |
| MSTRG.71291 | antisense | chr17:42026723-42027293:+ | 0.944173 | 6.598141 | 6.988274 | 0.002759 | 0.038047 |
| MSTRG.137019 | antisense | chr5:10492129-10498784:+ | 18.44898 | 110.8304 | 6.007398 | 0.002752 | 0.038077 |
| ENSG00000277825 | lincRNA | chr19:16352462-16353182:- | 0.340449 | 4.08177 | 11.98938 | 0.002792 | 0.038112 |
| MSTRG.11785 | linc | chr1:167580545-167588639:- | 34.78699 | 127.1408 | 3.654837 | 0.002815 | 0.038287 |
| MSTRG.53712 | antisense | chr14:64750701-64758924:+ | 247.1024 | 664.4388 | 2.688921 | 0.00285 | 0.038634 |
| MSTRG.57349 | linc | chr15:30420614-30427264:- | 32.83883 | 10.66977 | 0.324913 | 0.002867 | 0.03874 |
| ENSG00000267062 | antisense | chr19:12796823-12801849:+ | 13.12831 | 1.952869 | 0.148752 | 0.002946 | 0.039273 |
| ENSG00000281100 | TEC | chr3:36823151-36825158:- | 127.6909 | 307.8124 | 2.410605 | 0.002927 | 0.039289 |
| ENSG00000263958 | lincRNA | chr18:72868388-72881399:+ | 0.137833 | 3.520431 | 25.54136 | 0.002918 | 0.039302 |
| MSTRG.57603 | linc | chr15:35179487-35180820:- | 1.664889 | 8.373241 | 5.029309 | 0.002942 | 0.039354 |
| MSTRG.123759 | antisense | chr3:170025219-170066358:- | 13.50597 | 39.95595 | 2.958392 | 0.002972 | 0.03949 |
| MSTRG.151509 | antisense | chr6:42693633-42701330:+ | 228.0372 | 581.3446 | 2.549341 | 0.003008 | 0.039832 |
| MSTRG.150009 | linc | chr6:26670829-26686130:+ | 109.2233 | 45.59015 | 0.417403 | 0.003028 | 0.039839 |
| MSTRG.71880 | linc | chr17:47492723-47494007:+ | 0.330681 | 3.86497 | 11.6879 | 0.003023 | 0.039905 |
| MSTRG.43702 | linc | chr12:110712988-110714605:- | 6.067832 | 23.46822 | 3.867645 | 0.003075 | 0.040322 |
| MSTRG.115258 | linc | chr3:32513037-32515441:- | 60.31408 | 142.2186 | 2.357967 | 0.003096 | 0.040467 |
| ENSG00000278090 | lincRNA | chr15:99028538-99031053:+ | 5.003056 | 30.08905 | 6.014135 | 0.003149 | 0.040499 |
| ENSG00000272444 | lincRNA | chr14:101952416-101953063:+ | 0.316165 | 3.937847 | 12.45505 | 0.003124 | 0.040576 |
| MSTRG.193193 | linc | chrX:119612558-119613691:- | 4.387732 | 15.98747 | 3.643676 | 0.003145 | 0.040577 |
| MSTRG.1099 | linc | chr1:12212036-12524662:+ | 134.144 | 290.6678 | 2.166834 | 0.003116 | 0.040599 |
| MSTRG.3045 | antisense | chr1:36018418-36035297:- | 4.556418 | 17.07769 | 3.748052 | 0.003143 | 0.040692 |
| ENSG00000229191 | antisense | chr1:201023949-201028792:+ | 2.335146 | 12.4596 | 5.335682 | 0.003205 | 0.040963 |
| ENSG00000272037 | antisense | chr8:102256392-102257821:+ | 7.126676 | 23.9168 | 3.355954 | 0.003197 | 0.040995 |
| MSTRG.67138 | linc | chr16:84558374-84560708:- | 65.10892 | 298.4822 | 4.584351 | 0.003242 | 0.041299 |
| MSTRG.187195 | linc | chrX:1919802-1920351:- | 0.323862 | 4.295518 | 13.26342 | 0.003304 | 0.041559 |
| MSTRG.27474 | linc | chr11:14404580-14405880:- | 0.155297 | 3.135771 | 20.19205 | 0.003296 | 0.041598 |
| ENSG00000259548 | lincRNA | chr15:79832466-79833554:- | 9.753455 | 38.86247 | 3.984483 | 0.003289 | 0.041633 |
| MSTRG.14129 | linc | chr1:205080842-205083111:- | 10.79077 | 32.30892 | 2.994124 | 0.003287 | 0.041742 |
| MSTRG.53949 | linc | chr14:68856109-68859581:- | 10.34729 | 40.70267 | 3.933654 | 0.003348 | 0.041855 |
| ENSG00000266980 | antisense | chr17:75818815-75820055:- | 9.095932 | 27.3192 | 3.003453 | 0.003343 | 0.041928 |
| ENSG00000242770 | processed_transcript | chr3:112802478-112812819:+ | 6.748619 | 0.785457 | 0.116388 | 0.003404 | 0.04203 |
| ENSG00000238005 | lincRNA | chr1:234957342-234970062:- | 50.98942 | 158.8364 | 3.115086 | 0.003376 | 0.042073 |
| MSTRG.124639 | linc | chr3:183813638-183814751:- | 1.813351 | 8.808422 | 4.857538 | 0.003401 | 0.042123 |
| MSTRG.180364 | linc | chr9:63037907-63043047:- | 68.11563 | 12.7551 | 0.187257 | 0.003398 | 0.042217 |
| MSTRG.167571 | linc | chr7:149918931-149920510:- | 0.503389 | 4.360351 | 8.661998 | 0.003462 | 0.042613 |
| MSTRG.96741 | linc | chr2:172619919-172629363:+ | 59.50728 | 134.182 | 2.254884 | 0.003505 | 0.042755 |
| MSTRG.154591 | linc | chr6:108034922-108041033:- | 59.86929 | 138.9539 | 2.320954 | 0.003505 | 0.042878 |
| MSTRG.46436 | antisense | chr13:31189540-31200026:- | 175.9376 | 77.34903 | 0.439639 | 0.003501 | 0.042969 |
| MSTRG.16522 | linc | chr1:235916652-235917632:- | 0.348146 | 3.884028 | 11.15631 | 0.00358 | 0.043016 |
| MSTRG.144323 | linc | chr5:126852078-126866424:+ | 28.1245 | 72.33612 | 2.571997 | 0.003574 | 0.043075 |
| MSTRG.61364 | linc | chr15:84807049-84812566:+ | 74.8255 | 175.8949 | 2.350735 | 0.003558 | 0.043139 |
| MSTRG.169673 | linc | chr8:23522348-23522917:- | 0.331504 | 3.921505 | 11.82942 | 0.003574 | 0.043196 |
| MSTRG.29605 | linc | chr11:57523808-57540729:- | 156.5322 | 359.7037 | 2.297953 | 0.003554 | 0.043222 |
| MSTRG.1154 | linc | chr1:15673044-15675661:+ | 0.73593 | 6.547962 | 8.897536 | 0.003621 | 0.043382 |
| ENSG00000274515 | antisense | chr15:75645020-75645442:+ | 0.919011 | 6.230652 | 6.779734 | 0.003657 | 0.04368 |
| ENSG00000273565 | lincRNA | chr14:75176929-75177418:+ | 25.41126 | 6.758561 | 0.265967 | 0.003739 | 0.043744 |
| MSTRG.154253 | linc | chr6:105453557-105480939:+ | 220.593 | 64.62921 | 0.292979 | 0.003685 | 0.043751 |
| MSTRG.164384 | linc | chr7:76434027-76447321:- | 57.43622 | 135.3832 | 2.357105 | 0.003679 | 0.043806 |
| MSTRG.177323 | antisense | chr8:141326825-141327576:- | 0.325879 | 10.17012 | 31.20823 | 0.003737 | 0.043844 |
| MSTRG.223 | antisense | chr1:2501081-2503852:- | 40.86732 | 95.35897 | 2.333379 | 0.003769 | 0.043963 |
| MSTRG.139396 | linc | chr5:56641242-56726079:- | 2.39725 | 10.73795 | 4.479279 | 0.003736 | 0.043965 |
| MSTRG.168092 | linc | chr7:155787444-155788573:+ | 3.590502 | 19.05971 | 5.308369 | 0.00372 | 0.044037 |
| MSTRG.151070 | antisense | chr6:36240423-36248360:- | 53.37307 | 218.8522 | 4.100423 | 0.003731 | 0.044039 |
| MSTRG.39822 | antisense | chr12:57480489-57486959:+ | 396.9815 | 868.8559 | 2.188656 | 0.003799 | 0.044185 |
| MSTRG.69645 | antisense | chr17:20751128-20754372:+ | 4.50496 | 0.170809 | 0.037916 | 0.003828 | 0.044396 |
| ENSG00000237886 | antisense | chr9:136546212-136549893:+ | 10.94387 | 33.82319 | 3.090606 | 0.003841 | 0.044423 |
| MSTRG.53759 | linc | chr14:63765472-63786874:+ | 35.41126 | 287.2325 | 8.111333 | 0.003877 | 0.044704 |
| MSTRG.182303 | antisense | chr9:90973825-91007443:- | 74.96846 | 26.87035 | 0.358422 | 0.003908 | 0.044934 |
| MSTRG.107964 | antisense | chr21:33323679-33325423:- | 27.17251 | 72.79095 | 2.678846 | 0.003971 | 0.045527 |
| MSTRG.100318 | linc | chr2:218187313-218191342:+ | 1.992041 | 24.67445 | 12.38651 | 0.003983 | 0.045534 |
| MSTRG.20428 | antisense | chr10:12123852-12187967:+ | 43.21278 | 99.88746 | 2.311526 | 0.003995 | 0.045545 |
| ENSG00000267765 | antisense | chr17:42683187-42699466:- | 4.893218 | 17.90212 | 3.658558 | 0.004028 | 0.045661 |
| MSTRG.111991 | antisense | chr22:43162659-43163143:- | 0.840518 | 5.588341 | 6.648686 | 0.004043 | 0.045699 |
| MSTRG.13428 | linc | chr1:193496872-193505951:+ | 224.7541 | 90.41928 | 0.402303 | 0.004024 | 0.045747 |
| MSTRG.155006 | linc | chr6:114998119-115002631:+ | 22.04064 | 3.917199 | 0.177726 | 0.004061 | 0.045772 |
| MSTRG.83423 | antisense | chr19:41384144-41387737:+ | 19.19334 | 64.81227 | 3.37681 | 0.004094 | 0.046023 |
| MSTRG.27302 | antisense | chr11:11848936-11861282:+ | 1972.169 | 978.0614 | 0.495932 | 0.004149 | 0.046381 |
| ENSG00000270426 | sense_intronic | chr4:184471924-184472609:- | 52.59714 | 117.7011 | 2.237785 | 0.004144 | 0.046449 |
| ENSG00000277214 | lincRNA | chr16:79603572-79604177:+ | 10.78729 | 2.48257 | 0.230138 | 0.004203 | 0.046721 |
| MSTRG.30565 | linc | chr11:65574460-65575811:+ | 34.7843 | 84.69102 | 2.434749 | 0.004193 | 0.046742 |
| MSTRG.192676 | linc | chrX:107636256-107677157:- | 227.0111 | 466.143 | 2.053393 | 0.004218 | 0.04676 |
| MSTRG.184031 | antisense | chr9:110883509-110927695:- | 48.48198 | 104.7726 | 2.161063 | 0.004304 | 0.046808 |
| MSTRG.112444 | linc | chr22:49770626-49771153:- | 5.081274 | 20.95969 | 4.124888 | 0.004292 | 0.046809 |
| MSTRG.82708 | antisense | chr19:35323892-35419611:- | 18.80597 | 99.18168 | 5.273945 | 0.004269 | 0.046812 |
| MSTRG.94873 | linc | chr2:142831612-142871808:- | 265.0688 | 120.0909 | 0.453055 | 0.004324 | 0.046894 |
| MSTRG.91466 | antisense | chr2:85584843-85595867:- | 70.00307 | 160.8245 | 2.297392 | 0.004266 | 0.046901 |
| MSTRG.84281 | antisense | chr19:48681856-48693071:- | 0.471462 | 4.226322 | 8.964289 | 0.004292 | 0.046935 |
| ENSG00000230454 | lincRNA | chr3:50260303-50263358:+ | 57.91274 | 143.7878 | 2.482836 | 0.004262 | 0.046992 |
| MSTRG.185861 | antisense | chr9:133702737-133707270:+ | 8.779294 | 25.22243 | 2.872945 | 0.004258 | 0.047069 |
| MSTRG.20040 | linc | chr10:35581361-35587348:+ | 16.48529 | 47.02532 | 2.852563 | 0.004355 | 0.047105 |
| ENSG00000250437 | lincRNA | chr5:89581209-89677701:+ | 4.692698 | 23.04991 | 4.911868 | 0.004384 | 0.047296 |
| MSTRG.21186 | linc | chr10:46093349-46095534:- | 6.463952 | 20.35096 | 3.148377 | 0.004409 | 0.047436 |
| MSTRG.73235 | linc | chr17:67814820-67816335:- | 0.192849 | 3.33603 | 17.29868 | 0.004529 | 0.047954 |
| MSTRG.83071 | antisense | chr19:38734269-38735428:+ | 0.430962 | 5.058012 | 11.73655 | 0.004544 | 0.047986 |
| MSTRG.66237 | linc | chr16:69944860-69947735:+ | 34.97392 | 90.60186 | 2.590555 | 0.004526 | 0.048053 |
| MSTRG.111430 | antisense | chr22:37741031-37745984:- | 27.37337 | 80.15171 | 2.92809 | 0.004483 | 0.048107 |
| MSTRG.80667 | linc | chr19:8405802-8407678:+ | 6.933892 | 24.38797 | 3.517213 | 0.00457 | 0.048132 |
| MSTRG.44266 | antisense | chr12:118019020-118023386:- | 6.395058 | 20.55445 | 3.214114 | 0.00451 | 0.048132 |
| MSTRG.100997 | antisense | chr2:224973975-225299935:+ | 18.28335 | 3.763962 | 0.205868 | 0.004525 | 0.048168 |
| ENSG00000257495 | antisense | chr12:52601467-52615305:+ | 18.31595 | 47.44124 | 2.59016 | 0.004508 | 0.048242 |
| MSTRG.72000 | linc | chr17:49582996-49595808:- | 124.9774 | 263.9719 | 2.112157 | 0.004604 | 0.048365 |
| MSTRG.171940 | linc | chr8:65930911-65952743:- | 24.11851 | 71.73029 | 2.974077 | 0.004653 | 0.048377 |
| ENSG00000279259 | TEC | chr17:80147250-80148596:+ | 5.35181 | 21.58778 | 4.033735 | 0.004702 | 0.048383 |
| MSTRG.87758 | linc | chr2:28421598-28423235:+ | 14.00443 | 40.57633 | 2.897393 | 0.004666 | 0.048389 |
| MSTRG.179483 | antisense | chr9:35015555-35020180:- | 5.320555 | 17.60738 | 3.309312 | 0.004634 | 0.048432 |
| MSTRG.93889 | linc | chr2:122876101-122877120:+ | 4.839488 | 0.283175 | 0.058513 | 0.004647 | 0.048435 |
| MSTRG.80548 | antisense | chr19:8233377-8234022:+ | 0.137833 | 3.303529 | 23.9677 | 0.004697 | 0.048457 |
| MSTRG.163416 | linc | chr7:76320039-76322513:+ | 2.561417 | 14.27444 | 5.572866 | 0.00469 | 0.048512 |
| MSTRG.3064 | antisense | chr1:36359447-36366245:+ | 6.341469 | 26.3352 | 4.152855 | 0.004634 | 0.048555 |
| ENSG00000254789 | lincRNA | chr11:15571819-15622403:- | 6.961404 | 48.24672 | 6.930602 | 0.004739 | 0.048638 |
| MSTRG.43515 | linc | chr12:108607214-108612641:- | 62.33651 | 172.3541 | 2.764898 | 0.004781 | 0.048941 |
| MSTRG.13753 | antisense | chr1:200387442-200397631:- | 193.8603 | 553.1864 | 2.853531 | 0.004811 | 0.049121 |

Table S2. The information of 311 significantly differentially expressed T1D- mRNAs and healthy control.

| **Gene** | **T1D**  **normalize** | **Control**  **normalize** | **Fold**  **Change** | **p-value** | **FDR** |
| --- | --- | --- | --- | --- | --- |
| ENSG00000177606 | 290.6111 | 3199.693 | 0.090825 | 2.27E-18 | 5E-15 |
| ENSG00000113070 | 34.90895 | 400.0398 | 0.087264 | 8.75E-15 | 9.65E-12 |
| ENSG00000119508 | 27.37751 | 326.6264 | 0.083819 | 2.03E-13 | 1.49E-10 |
| ENSG00000124882 | 19.03672 | 208.071 | 0.091491 | 4.57E-10 | 2.02E-07 |
| ENSG00000153234 | 279.3861 | 1454.197 | 0.192124 | 4.48E-10 | 2.47E-07 |
| ENSG00000172179 | 0.941145 | 33.75164 | 0.027884 | 1.86E-09 | 6.83E-07 |
| ENSG00000003137 | 17.77552 | 0.31054 | 57.24073 | 2.65E-09 | 8.34E-07 |
| ENSG00000060709 | 28.64434 | 1.449632 | 19.75973 | 3.89E-09 | 1.07E-06 |
| ENSG00000162772 | 42.23578 | 284.0244 | 0.148705 | 5.07E-09 | 1.24E-06 |
| ENSG00000123358 | 103.1214 | 913.2576 | 0.112916 | 1.18E-08 | 2.6E-06 |
| ENSG00000167034 | 127.2693 | 20.86977 | 6.09826 | 2.50E-08 | 5E-06 |
| ENSG00000154640 | 118.6247 | 475.1633 | 0.24965 | 3.21E-08 | 5.91E-06 |
| ENSG00000235961 | 29.70577 | 2.127212 | 13.96465 | 3.52E-08 | 5.97E-06 |
| ENSG00000132639 | 0.633402 | 26.9034 | 0.023544 | 3.84E-08 | 6.05E-06 |
| ENSG00000155090 | 876.7618 | 3431.942 | 0.255471 | 4.12E-08 | 6.06E-06 |
| ENSG00000182752 | 0.443396 | 15.15348 | 0.02926 | 1.32E-07 | 1.82E-05 |
| ENSG00000162069 | 33.6617 | 3.020198 | 11.14553 | 4.92E-07 | 6.39E-05 |
| ENSG00000158050 | 300.3857 | 1038.517 | 0.289245 | 7.12E-07 | 8.72E-05 |
| ENSG00000134321 | 1336.029 | 405.0456 | 3.298466 | 8.26E-07 | 9.59E-05 |
| ENSG00000185697 | 1203.605 | 3465.989 | 0.347262 | 9.81E-07 | 0.000108 |
| ENSG00000281106 | 586.5463 | 177.0759 | 3.3124 | 2.59E-06 | 0.000272 |
| ENSG00000104723 | 0.996358 | 20.64047 | 0.048272 | 3.17E-06 | 0.000304 |
| ENSG00000137869 | 0.288294 | 11.44492 | 0.02519 | 3.05E-06 | 0.000306 |
| ENSG00000188186 | 1001.059 | 371.4703 | 2.694855 | 5.14E-06 | 0.000473 |
| ENSG00000071909 | 18.96489 | 92.29041 | 0.205492 | 6.04E-06 | 0.000532 |
| ENSG00000204482 | 2635.648 | 1014.824 | 2.597148 | 7.22E-06 | 0.000612 |
| ENSG00000164120 | 207.0587 | 583.592 | 0.354801 | 7.53E-06 | 0.000615 |
| ENSG00000205220 | 304.386 | 95.25036 | 3.195641 | 8.03E-06 | 0.000632 |
| ENSG00000168062 | 148.5848 | 27.81975 | 5.340982 | 1.24E-05 | 0.000915 |
| ENSG00000092758 | 139.6098 | 42.21762 | 3.306908 | 1.24E-05 | 0.000942 |
| ENSG00000259332 | 55.97254 | 11.62771 | 4.813718 | 1.56E-05 | 0.001107 |
| ENSG00000002919 | 791.688 | 307.5769 | 2.573951 | 1.62E-05 | 0.001119 |
| ENSG00000142178 | 40.94765 | 145.366 | 0.281686 | 2.24E-05 | 0.001493 |
| ENSG00000111537 | 37.87736 | 157.5372 | 0.240434 | 2.32E-05 | 0.001507 |
| ENSG00000196154 | 4292.915 | 1734.755 | 2.474652 | 2.55E-05 | 0.001609 |
| ENSG00000187608 | 604.2608 | 218.0445 | 2.771272 | 2.79E-05 | 0.001661 |
| ENSG00000187624 | 16.22426 | 2.19837 | 7.380135 | 2.77E-05 | 0.001695 |
| ENSG00000100234 | 1.364403 | 15.77698 | 0.086481 | 3.33E-05 | 0.001789 |
| ENSG00000198918 | 4077.943 | 1337.917 | 3.04798 | 3.32E-05 | 0.001829 |
| ENSG00000125910 | 2099.557 | 855.5999 | 2.453901 | 3.26E-05 | 0.001843 |
| ENSG00000180914 | 21.63757 | 84.22601 | 0.256899 | 3.22E-05 | 0.001866 |
| ENSG00000172985 | 159.9409 | 49.19322 | 3.25128 | 3.64E-05 | 0.001869 |
| ENSG00000229314 | 64.09622 | 6.865873 | 9.335479 | 3.63E-05 | 0.001907 |
| ENSG00000197956 | 5111.024 | 2035.945 | 2.510394 | 3.96E-05 | 0.001983 |
| ENSG00000213741 | 2794.095 | 1008.76 | 2.769833 | 4.10E-05 | 0.002011 |
| ENSG00000196411 | 221.1217 | 64.99696 | 3.402031 | 4.43E-05 | 0.002125 |
| ENSG00000167244 | 0.492629 | 9.967346 | 0.049424 | 4.53E-05 | 0.002127 |
| ENSG00000010030 | 147.4958 | 41.24859 | 3.575779 | 4.82E-05 | 0.002216 |
| ENSG00000161835 | 85.53846 | 321.6492 | 0.265937 | 5.09E-05 | 0.002289 |
| ENSG00000213654 | 5133.208 | 2170.72 | 2.364749 | 5.80E-05 | 0.00256 |
| ENSG00000150687 | 247.4737 | 638.2904 | 0.387713 | 6.51E-05 | 0.002762 |
| ENSG00000205927 | 15.783 | 1.566719 | 10.07392 | 6.69E-05 | 0.002782 |
| ENSG00000148180 | 288.885 | 106.258 | 2.718711 | 6.49E-05 | 0.002806 |
| ENSG00000149131 | 356.6347 | 114.2346 | 3.121949 | 7.36E-05 | 0.003007 |
| ENSG00000154165 | 101.2089 | 413.1194 | 0.244987 | 8.57E-05 | 0.003435 |
| ENSG00000137267 | 10.92002 | 59.50254 | 0.183522 | 8.92E-05 | 0.003451 |
| ENSG00000172967 | 13.08631 | 1.625786 | 8.049218 | 8.82E-05 | 0.003473 |
| ENSG00000006327 | 97.09395 | 16.98625 | 5.716031 | 0.000104 | 0.003968 |
| ENSG00000218819 | 6.500393 | 33.25638 | 0.195463 | 0.000111 | 0.004148 |
| ENSG00000034510 | 7840.2 | 3197.871 | 2.451694 | 0.000119 | 0.004358 |
| ENSG00000125740 | 982.1381 | 3240.591 | 0.303074 | 0.000126 | 0.004549 |
| ENSG00000182117 | 720.3658 | 279.9661 | 2.573047 | 0.000148 | 0.005248 |
| ENSG00000069702 | 1337.744 | 2948.299 | 0.453734 | 0.000169 | 0.005831 |
| ENSG00000148488 | 71.32554 | 190.3883 | 0.374632 | 0.000168 | 0.005887 |
| ENSG00000117984 | 1237.419 | 550.9514 | 2.245968 | 0.000181 | 0.006133 |
| ENSG00000163564 | 1090.543 | 2415.737 | 0.451433 | 0.000202 | 0.00676 |
| ENSG00000157856 | 8.631439 | 0.840573 | 10.26852 | 0.000209 | 0.006894 |
| ENSG00000197057 | 447.3535 | 1238.778 | 0.361125 | 0.000218 | 0.006965 |
| ENSG00000152583 | 0.156234 | 6.526152 | 0.02394 | 0.000218 | 0.007063 |
| ENSG00000116667 | 362.8566 | 825.8311 | 0.439384 | 0.000232 | 0.007219 |
| ENSG00000125968 | 8.969364 | 66.39168 | 0.135098 | 0.00023 | 0.007242 |
| ENSG00000282608 | 35.23037 | 8.003246 | 4.402011 | 0.000244 | 0.007284 |
| ENSG00000177191 | 453.4686 | 96.16015 | 4.715765 | 0.000241 | 0.00729 |
| ENSG00000138166 | 197.0296 | 484.5653 | 0.406611 | 0.00024 | 0.007364 |
| ENSG00000137393 | 2796.271 | 1255.23 | 2.227697 | 0.000252 | 0.007405 |
| ENSG00000163751 | 57.72771 | 168.9315 | 0.341723 | 0.000256 | 0.007439 |
| ENSG00000109771 | 44.80112 | 129.8338 | 0.345065 | 0.00028 | 0.00801 |
| ENSG00000139116 | 375.052 | 828.9937 | 0.452418 | 0.000283 | 0.008012 |
| ENSG00000197837 | 811.9878 | 347.2526 | 2.338321 | 0.000318 | 0.008869 |
| ENSG00000125657 | 5.211202 | 24.48272 | 0.212852 | 0.000346 | 0.009532 |
| ENSG00000143507 | 183.0649 | 462.9081 | 0.395467 | 0.000358 | 0.00974 |
| ENSG00000169442 | 3207.556 | 1335.443 | 2.401866 | 0.00037 | 0.009956 |
| ENSG00000129116 | 141.8605 | 326.0266 | 0.43512 | 0.000391 | 0.010267 |
| ENSG00000135114 | 801.1729 | 308.1968 | 2.59955 | 0.000388 | 0.010301 |
| ENSG00000214212 | 801.1177 | 372.2718 | 2.15197 | 0.000411 | 0.01066 |
| ENSG00000109472 | 1.07262 | 9.495583 | 0.11296 | 0.00043 | 0.011015 |
| ENSG00000136826 | 580.0111 | 1433.107 | 0.404723 | 0.000452 | 0.011468 |
| ENSG00000125812 | 312.9679 | 760.3246 | 0.411624 | 0.000497 | 0.012307 |
| ENSG00000197061 | 890.2156 | 364.7542 | 2.44059 | 0.000492 | 0.012327 |
| ENSG00000134871 | 2.303184 | 18.79504 | 0.122542 | 0.00052 | 0.012599 |
| ENSG00000182253 | 205.0782 | 573.7406 | 0.357441 | 0.000519 | 0.012727 |
| ENSG00000129595 | 93.36839 | 238.41 | 0.39163 | 0.000534 | 0.012799 |
| ENSG00000137941 | 56.17453 | 147.1658 | 0.381709 | 0.000559 | 0.013107 |
| ENSG00000184361 | 19.53486 | 4.103049 | 4.761058 | 0.000573 | 0.013156 |
| ENSG00000146674 | 41.69413 | 114.0917 | 0.365444 | 0.000556 | 0.013178 |
| ENSG00000196586 | 111.4281 | 320.3416 | 0.347841 | 0.000571 | 0.013243 |
| ENSG00000124588 | 1012.065 | 386.5629 | 2.618112 | 0.0006 | 0.013646 |
| ENSG00000198502 | 550.648 | 16.99531 | 32.39999 | 0.000616 | 0.013862 |
| ENSG00000156453 | 31.31798 | 90.73138 | 0.345173 | 0.000629 | 0.014008 |
| ENSG00000110848 | 871.851 | 3898.772 | 0.223622 | 0.000638 | 0.014075 |
| ENSG00000160991 | 2552.181 | 1237.471 | 2.062417 | 0.000651 | 0.014202 |
| ENSG00000165131 | 11.61171 | 1.875189 | 6.192287 | 0.000676 | 0.014334 |
| ENSG00000162881 | 169.3708 | 48.65988 | 3.480707 | 0.000675 | 0.014457 |
| ENSG00000138778 | 136.8828 | 361.6258 | 0.378521 | 0.000674 | 0.01458 |
| ENSG00000135052 | 185.7941 | 454.131 | 0.40912 | 0.000699 | 0.01468 |
| ENSG00000092067 | 55.61941 | 18.89906 | 2.942972 | 0.000738 | 0.015071 |
| ENSG00000205090 | 8.156474 | 0.980423 | 8.319342 | 0.000734 | 0.015132 |
| ENSG00000100453 | 645.9317 | 1314.413 | 0.491422 | 0.00073 | 0.015191 |
| ENSG00000213931 | 30.87515 | 2.133531 | 14.47138 | 0.00078 | 0.015353 |
| ENSG00000092036 | 310.827 | 130.0577 | 2.389917 | 0.000805 | 0.015428 |
| ENSG00000113448 | 1303.292 | 2713.148 | 0.480361 | 0.000777 | 0.015444 |
| ENSG00000052126 | 154.4392 | 351.7608 | 0.439046 | 0.000793 | 0.015468 |
| ENSG00000168298 | 3107.371 | 10966.7 | 0.283346 | 0.0008 | 0.015473 |
| ENSG00000205352 | 1278.458 | 617.6123 | 2.070001 | 0.000766 | 0.015496 |
| ENSG00000162413 | 1271.96 | 623.0881 | 2.04138 | 0.000775 | 0.015527 |
| ENSG00000148019 | 1113.131 | 2230.022 | 0.499157 | 0.000827 | 0.01559 |
| ENSG00000135338 | 15.44563 | 50.83876 | 0.303816 | 0.000822 | 0.015632 |
| ENSG00000163993 | 430.3129 | 72.52541 | 5.933271 | 0.000875 | 0.01635 |
| ENSG00000239732 | 11.76123 | 1.964451 | 5.987031 | 0.000896 | 0.016609 |
| ENSG00000085117 | 1213.33 | 602.7326 | 2.013048 | 0.000906 | 0.016652 |
| ENSG00000183307 | 319.1628 | 106.1639 | 3.006322 | 0.00098 | 0.017857 |
| ENSG00000133773 | 712.8755 | 1491.583 | 0.477932 | 0.001019 | 0.018111 |
| ENSG00000255819 | 178.4487 | 395.2927 | 0.451434 | 0.001018 | 0.018258 |
| ENSG00000173542 | 1861.241 | 4225.506 | 0.440478 | 0.001014 | 0.018326 |
| ENSG00000205609 | 219.9765 | 570.4744 | 0.385603 | 0.001057 | 0.018499 |
| ENSG00000175938 | 115.9536 | 45.70659 | 2.536912 | 0.00105 | 0.018522 |
| ENSG00000186648 | 32.5346 | 87.3125 | 0.372623 | 0.001089 | 0.0189 |
| ENSG00000133874 | 402.7761 | 180.9864 | 2.22545 | 0.001112 | 0.019015 |
| ENSG00000174944 | 473.448 | 212.1327 | 2.231848 | 0.001111 | 0.019134 |
| ENSG00000179841 | 56.48711 | 155.9379 | 0.362241 | 0.001173 | 0.019743 |
| ENSG00000198108 | 2.891642 | 23.91998 | 0.120888 | 0.001172 | 0.019874 |
| ENSG00000135828 | 1076.921 | 537.6683 | 2.002948 | 0.001193 | 0.019928 |
| ENSG00000109339 | 8.284403 | 31.7422 | 0.26099 | 0.001213 | 0.020102 |
| ENSG00000182899 | 6778.637 | 2950.22 | 2.297672 | 0.001258 | 0.0204 |
| ENSG00000080822 | 1088.399 | 2241.241 | 0.485624 | 0.001271 | 0.020452 |
| ENSG00000165244 | 154.783 | 377.3815 | 0.41015 | 0.001257 | 0.020536 |
| ENSG00000144460 | 1.029638 | 9.473943 | 0.108681 | 0.00125 | 0.020574 |
| ENSG00000137261 | 182.4921 | 62.77724 | 2.906978 | 0.0013 | 0.020777 |
| ENSG00000173153 | 513.0983 | 248.3621 | 2.065929 | 0.001319 | 0.020779 |
| ENSG00000105767 | 49.00001 | 11.32232 | 4.327736 | 0.001311 | 0.020797 |
| ENSG00000232810 | 323.6692 | 155.1551 | 2.086101 | 0.00135 | 0.021107 |
| ENSG00000125356 | 372.0107 | 158.3323 | 2.349556 | 0.001382 | 0.021457 |
| ENSG00000167261 | 2563.994 | 987.9875 | 2.595168 | 0.001421 | 0.021909 |
| ENSG00000143546 | 29487.45 | 10108.68 | 2.917042 | 0.001485 | 0.022132 |
| ENSG00000130522 | 831.4414 | 1677.331 | 0.495693 | 0.001448 | 0.022167 |
| ENSG00000179564 | 6.768199 | 0.70956 | 9.53859 | 0.00148 | 0.022206 |
| ENSG00000102886 | 152.0331 | 62.94598 | 2.415295 | 0.001477 | 0.022313 |
| ENSG00000165195 | 361.7684 | 748.7985 | 0.483132 | 0.001469 | 0.022346 |
| ENSG00000144724 | 12.59391 | 43.53225 | 0.289301 | 0.00152 | 0.022495 |
| ENSG00000119042 | 25.08042 | 69.78815 | 0.359379 | 0.001531 | 0.022505 |
| ENSG00000172159 | 415.7815 | 1269.061 | 0.327629 | 0.001577 | 0.022579 |
| ENSG00000126524 | 632.6741 | 1293.969 | 0.488941 | 0.001573 | 0.022671 |
| ENSG00000049130 | 21.8727 | 75.96601 | 0.287927 | 0.001569 | 0.022757 |
| ENSG00000205189 | 657.3714 | 1403.75 | 0.468297 | 0.001564 | 0.022843 |
| ENSG00000167768 | 0.960444 | 9.761355 | 0.098393 | 0.001646 | 0.023415 |
| ENSG00000064225 | 736.0143 | 358.3753 | 2.053753 | 0.001728 | 0.024424 |
| ENSG00000187808 | 103.9203 | 40.33529 | 2.576412 | 0.001783 | 0.024577 |
| ENSG00000165929 | 3627.336 | 7504.979 | 0.483324 | 0.001767 | 0.02466 |
| ENSG00000041515 | 15.7385 | 54.76582 | 0.287378 | 0.001782 | 0.024715 |
| ENSG00000126709 | 861.3876 | 400.7076 | 2.149666 | 0.00176 | 0.024723 |
| ENSG00000163435 | 54.26912 | 17.62873 | 3.078447 | 0.001807 | 0.024747 |
| ENSG00000118276 | 101.7976 | 219.7186 | 0.463309 | 0.001878 | 0.025255 |
| ENSG00000235453 | 89.72253 | 34.34423 | 2.612448 | 0.001873 | 0.025331 |
| ENSG00000185745 | 2593.122 | 641.3249 | 4.043383 | 0.001861 | 0.025332 |
| ENSG00000090104 | 191.6516 | 817.2981 | 0.234494 | 0.001906 | 0.025473 |
| ENSG00000247596 | 471.2164 | 233.067 | 2.021807 | 0.001961 | 0.02605 |
| ENSG00000172058 | 13.7219 | 3.06088 | 4.482994 | 0.002012 | 0.026251 |
| ENSG00000128791 | 305.8393 | 692.6793 | 0.441531 | 0.002003 | 0.026295 |
| ENSG00000050628 | 26.05352 | 75.24899 | 0.346231 | 0.001998 | 0.026384 |
| ENSG00000225873 | 142.8866 | 33.07947 | 4.319496 | 0.002044 | 0.026515 |
| ENSG00000233822 | 235.1589 | 536.6895 | 0.438166 | 0.00209 | 0.026944 |
| ENSG00000183508 | 1375.637 | 2777.379 | 0.495301 | 0.002147 | 0.027524 |
| ENSG00000119471 | 1682.671 | 779.3683 | 2.159019 | 0.002169 | 0.027644 |
| ENSG00000154803 | 474.604 | 233.1744 | 2.035404 | 0.002186 | 0.027707 |
| ENSG00000163535 | 103.9553 | 227.9385 | 0.456067 | 0.002319 | 0.02889 |
| ENSG00000135407 | 456.2398 | 119.0903 | 3.831041 | 0.002294 | 0.02891 |
| ENSG00000250510 | 176.0975 | 81.78816 | 2.153093 | 0.002312 | 0.028969 |
| ENSG00000137767 | 485.6594 | 236.7604 | 2.051269 | 0.002339 | 0.028978 |
| ENSG00000184371 | 451.4314 | 142.5625 | 3.16655 | 0.00236 | 0.02907 |
| ENSG00000248712 | 37.84292 | 12.65635 | 2.990034 | 0.002378 | 0.029128 |
| ENSG00000163221 | 1250.189 | 480.998 | 2.599156 | 0.002431 | 0.029454 |
| ENSG00000126432 | 747.7884 | 364.3109 | 2.052611 | 0.00243 | 0.029607 |
| ENSG00000182489 | 58.46997 | 21.30257 | 2.744737 | 0.002577 | 0.029907 |
| ENSG00000229117 | 14475.63 | 5944.356 | 2.43519 | 0.002482 | 0.029908 |
| ENSG00000114268 | 1551.847 | 507.6728 | 3.056786 | 0.002568 | 0.029957 |
| ENSG00000125900 | 98.69865 | 33.8948 | 2.911911 | 0.002506 | 0.030028 |
| ENSG00000155330 | 203.3527 | 420.0941 | 0.484065 | 0.002563 | 0.030058 |
| ENSG00000183762 | 762.5613 | 153.064 | 4.981976 | 0.002609 | 0.030122 |
| ENSG00000170962 | 173.7268 | 358.1447 | 0.485074 | 0.002555 | 0.030122 |
| ENSG00000112742 | 21.72657 | 64.14849 | 0.338692 | 0.002542 | 0.030137 |
| ENSG00000260286 | 85.61477 | 32.96092 | 2.597463 | 0.002536 | 0.030221 |
| ENSG00000163959 | 9.772956 | 2.091073 | 4.673657 | 0.002641 | 0.030332 |
| ENSG00000002726 | 35.02711 | 3.784323 | 9.255845 | 0.002704 | 0.030736 |
| ENSG00000103056 | 270.9478 | 103.1348 | 2.627124 | 0.002702 | 0.030873 |
| ENSG00000136527 | 5205.703 | 10727.2 | 0.485281 | 0.002747 | 0.031061 |
| ENSG00000109743 | 1334.273 | 656.3216 | 2.032956 | 0.002771 | 0.03117 |
| ENSG00000025039 | 728.8131 | 326.7966 | 2.230173 | 0.002785 | 0.031171 |
| ENSG00000135116 | 63.25893 | 20.20763 | 3.130449 | 0.002806 | 0.031249 |
| ENSG00000008438 | 277.2521 | 71.80555 | 3.861151 | 0.002851 | 0.031272 |
| ENSG00000072133 | 2.357858 | 11.65014 | 0.202389 | 0.002835 | 0.031415 |
| ENSG00000223609 | 54.00756 | 8.635231 | 6.254328 | 0.00285 | 0.031426 |
| ENSG00000205809 | 104.2681 | 261.4342 | 0.398831 | 0.002894 | 0.031585 |
| ENSG00000068001 | 78.20294 | 31.1605 | 2.509682 | 0.002936 | 0.031739 |
| ENSG00000152766 | 108.3571 | 41.13783 | 2.634003 | 0.002925 | 0.031775 |
| ENSG00000163660 | 9652.187 | 20437.74 | 0.472273 | 0.003065 | 0.032971 |
| ENSG00000276203 | 5.948954 | 24.55996 | 0.242222 | 0.003181 | 0.033085 |
| ENSG00000138182 | 536.5579 | 1144.541 | 0.468797 | 0.003113 | 0.033163 |
| ENSG00000256812 | 61.65713 | 19.01369 | 3.242775 | 0.0031 | 0.033183 |
| ENSG00000158485 | 0.75099 | 6.966187 | 0.107805 | 0.003178 | 0.033206 |
| ENSG00000198673 | 619.3449 | 275.6553 | 2.246809 | 0.003173 | 0.033314 |
| ENSG00000105048 | 36.83047 | 11.99402 | 3.070736 | 0.003168 | 0.033422 |
| ENSG00000102755 | 124.9644 | 53.32192 | 2.343584 | 0.003158 | 0.033475 |
| ENSG00000175305 | 20.24871 | 57.52569 | 0.351994 | 0.003253 | 0.033671 |
| ENSG00000006468 | 1.381242 | 8.969514 | 0.153993 | 0.003281 | 0.033811 |
| ENSG00000123836 | 590.6324 | 284.4639 | 2.0763 | 0.003324 | 0.034088 |
| ENSG00000105404 | 630.8831 | 314.3332 | 2.007052 | 0.003356 | 0.034096 |
| ENSG00000174326 | 18.31449 | 4.986011 | 3.673174 | 0.003342 | 0.034116 |
| ENSG00000277157 | 288.5549 | 125.1089 | 2.306429 | 0.003409 | 0.034478 |
| ENSG00000269858 | 847.1213 | 383.3331 | 2.209883 | 0.003427 | 0.034503 |
| ENSG00000073737 | 667.8203 | 195.8313 | 3.410181 | 0.003521 | 0.035129 |
| ENSG00000125735 | 739.758 | 222.8724 | 3.3192 | 0.003518 | 0.03526 |
| ENSG00000177663 | 9679.093 | 4679.546 | 2.068383 | 0.003584 | 0.03528 |
| ENSG00000147036 | 108.0408 | 300.087 | 0.360031 | 0.003606 | 0.035334 |
| ENSG00000177398 | 15.91907 | 4.563018 | 3.488716 | 0.003577 | 0.035367 |
| ENSG00000055732 | 6.936316 | 24.68124 | 0.281036 | 0.003567 | 0.035425 |
| ENSG00000197756 | 6727.047 | 3126.418 | 2.151679 | 0.003634 | 0.035454 |
| ENSG00000136634 | 2.256862 | 15.15619 | 0.148907 | 0.003667 | 0.035622 |
| ENSG00000279483 | 19.25318 | 5.583541 | 3.448202 | 0.003717 | 0.035944 |
| ENSG00000061676 | 272.1035 | 807.2614 | 0.33707 | 0.003792 | 0.036513 |
| ENSG00000177359 | 24.38092 | 158.6247 | 0.153702 | 0.003813 | 0.036557 |
| ENSG00000168772 | 20.32679 | 59.08006 | 0.344055 | 0.003877 | 0.037009 |
| ENSG00000151725 | 48.87458 | 112.7212 | 0.433588 | 0.003944 | 0.037486 |
| ENSG00000131016 | 92.58111 | 229.0912 | 0.404123 | 0.003984 | 0.037702 |
| ENSG00000162614 | 314.8555 | 1213.91 | 0.259373 | 0.004011 | 0.037797 |
| ENSG00000169902 | 381.3956 | 133.6061 | 2.854626 | 0.004087 | 0.038345 |
| ENSG00000158825 | 1004.479 | 361.65 | 2.77749 | 0.004171 | 0.038804 |
| ENSG00000134853 | 9.040034 | 30.37409 | 0.297623 | 0.004154 | 0.038812 |
| ENSG00000178053 | 9.867713 | 31.29386 | 0.315324 | 0.004235 | 0.039069 |
| ENSG00000050030 | 20.50452 | 51.198 | 0.400494 | 0.004231 | 0.0392 |
| ENSG00000116299 | 1653.689 | 555.3051 | 2.977983 | 0.004314 | 0.039637 |
| ENSG00000214530 | 299.299 | 126.5535 | 2.364999 | 0.004369 | 0.039641 |
| ENSG00000157087 | 1.233032 | 7.549826 | 0.163319 | 0.004336 | 0.039674 |
| ENSG00000172780 | 107.1594 | 35.00581 | 3.061188 | 0.004365 | 0.039771 |
| ENSG00000100024 | 102.99 | 39.53244 | 2.605202 | 0.004412 | 0.039871 |
| ENSG00000154479 | 1.528321 | 11.11718 | 0.137474 | 0.004587 | 0.041112 |
| ENSG00000131469 | 5119.54 | 2452.064 | 2.08785 | 0.004578 | 0.041204 |
| ENSG00000275993 | 61.52765 | 162.6733 | 0.378228 | 0.004646 | 0.04148 |
| ENSG00000204345 | 26.20463 | 8.638378 | 3.033512 | 0.004731 | 0.042068 |
| ENSG00000198796 | 13.09058 | 39.91829 | 0.327934 | 0.004757 | 0.042129 |
| ENSG00000166148 | 91.48056 | 318.7339 | 0.287012 | 0.004886 | 0.042921 |
| ENSG00000074660 | 679.3986 | 212.6468 | 3.194962 | 0.004867 | 0.042923 |
| ENSG00000174917 | 194.2579 | 86.96795 | 2.233672 | 0.004926 | 0.043104 |
| ENSG00000105967 | 1023.067 | 2085.966 | 0.490452 | 0.004966 | 0.043278 |
| ENSG00000187122 | 45.96265 | 16.40254 | 2.802167 | 0.005019 | 0.043396 |
| ENSG00000152213 | 744.5417 | 336.9126 | 2.209895 | 0.005 | 0.04341 |
| ENSG00000125753 | 7285.718 | 3031.506 | 2.403333 | 0.00513 | 0.043844 |
| ENSG00000124134 | 15.14266 | 2.795186 | 5.417407 | 0.005126 | 0.043983 |
| ENSG00000276368 | 182.3896 | 531.7659 | 0.342988 | 0.005108 | 0.044 |
| ENSG00000160593 | 13968.42 | 5754.778 | 2.427274 | 0.005214 | 0.04439 |
| ENSG00000133739 | 109.5391 | 224.8926 | 0.487073 | 0.005264 | 0.044469 |
| ENSG00000112964 | 0.155791 | 3.798473 | 0.041014 | 0.005258 | 0.044591 |
| ENSG00000197361 | 30.10194 | 11.02994 | 2.729111 | 0.005457 | 0.044734 |
| ENSG00000170956 | 1694.277 | 489.3991 | 3.461954 | 0.005456 | 0.044889 |
| ENSG00000173482 | 198.9333 | 400.3322 | 0.496921 | 0.00543 | 0.045012 |
| ENSG00000280360 | 0.156234 | 4.274354 | 0.036551 | 0.005451 | 0.045018 |
| ENSG00000115290 | 27.47333 | 92.54049 | 0.296879 | 0.005418 | 0.045085 |
| ENSG00000121933 | 23.00835 | 6.583682 | 3.494755 | 0.005521 | 0.045087 |
| ENSG00000107863 | 1239.79 | 3178.545 | 0.39005 | 0.005408 | 0.045167 |
| ENSG00000129450 | 1619.487 | 708.0349 | 2.287299 | 0.005369 | 0.045184 |
| ENSG00000142089 | 3790.482 | 1061.854 | 3.569683 | 0.005402 | 0.045291 |
| ENSG00000121552 | 1005.835 | 469.862 | 2.140702 | 0.005593 | 0.045339 |
| ENSG00000164509 | 21.65203 | 56.92696 | 0.380348 | 0.005586 | 0.045448 |
| ENSG00000090238 | 3134.865 | 1434.943 | 2.184661 | 0.005734 | 0.045479 |
| ENSG00000184304 | 2.131087 | 11.62496 | 0.18332 | 0.005778 | 0.045502 |
| ENSG00000136371 | 88.91982 | 32.50464 | 2.735604 | 0.005758 | 0.045509 |
| ENSG00000183844 | 45.52862 | 1.571541 | 28.9707 | 0.005641 | 0.045558 |
| ENSG00000178226 | 26.64179 | 8.756134 | 3.042643 | 0.005731 | 0.045621 |
| ENSG00000131459 | 6.360318 | 23.61685 | 0.269313 | 0.00573 | 0.045774 |
| ENSG00000157350 | 2207.821 | 1078.82 | 2.046514 | 0.005725 | 0.045901 |
| ENSG00000140995 | 1732.641 | 817.9617 | 2.118242 | 0.005706 | 0.045922 |
| ENSG00000091262 | 89.76017 | 38.5965 | 2.325604 | 0.005863 | 0.046004 |
| ENSG00000253873 | 5.485642 | 19.30461 | 0.284162 | 0.005989 | 0.046667 |
| ENSG00000102796 | 494.7143 | 246.0742 | 2.010428 | 0.006082 | 0.04673 |
| ENSG00000123700 | 2072.772 | 518.0686 | 4.000961 | 0.006109 | 0.04677 |
| ENSG00000111671 | 161.1557 | 71.08977 | 2.266932 | 0.005988 | 0.046821 |
| ENSG00000176834 | 77.28959 | 31.11277 | 2.484176 | 0.006075 | 0.046834 |
| ENSG00000133067 | 94.69477 | 196.3699 | 0.482227 | 0.006063 | 0.046908 |
| ENSG00000253305 | 10.92945 | 30.95682 | 0.353055 | 0.006057 | 0.047024 |
| ENSG00000103226 | 309.8655 | 115.9945 | 2.67138 | 0.006183 | 0.047177 |
| ENSG00000151789 | 75.4331 | 247.8811 | 0.304312 | 0.006211 | 0.047227 |
| ENSG00000160588 | 1753.977 | 648.6659 | 2.703975 | 0.006331 | 0.047325 |
| ENSG00000134020 | 7.590718 | 1.662144 | 4.566824 | 0.006356 | 0.047348 |
| ENSG00000172243 | 9704.533 | 3960.878 | 2.450096 | 0.00625 | 0.047355 |
| ENSG00000156395 | 18.75146 | 46.03774 | 0.407306 | 0.006401 | 0.047363 |
| ENSG00000260861 | 19.94551 | 6.508347 | 3.064605 | 0.006324 | 0.047426 |
| ENSG00000177700 | 176.0079 | 84.13072 | 2.092077 | 0.006285 | 0.047463 |
| ENSG00000075290 | 4.59117 | 0.458195 | 10.02013 | 0.00631 | 0.04749 |
| ENSG00000131203 | 40.41785 | 14.85367 | 2.721068 | 0.0064 | 0.047513 |
| ENSG00000217555 | 216.9904 | 90.62499 | 2.394377 | 0.006581 | 0.047889 |
| ENSG00000110042 | 376.4863 | 185.5697 | 2.028814 | 0.006562 | 0.047909 |
| ENSG00000181220 | 2295.944 | 1105.654 | 2.076548 | 0.00655 | 0.047983 |
| ENSG00000274180 | 1130.684 | 399.1216 | 2.832931 | 0.006549 | 0.048137 |
| ENSG00000112715 | 218.4777 | 472.1191 | 0.46276 | 0.006715 | 0.04823 |
| ENSG00000172183 | 2583.585 | 1257.673 | 2.054259 | 0.006541 | 0.048237 |
| ENSG00000150045 | 525.4869 | 1069.25 | 0.491453 | 0.006714 | 0.048378 |
| ENSG00000140932 | 790.5025 | 150.672 | 5.246512 | 0.006711 | 0.04852 |
| ENSG00000189127 | 128.2323 | 45.55812 | 2.814697 | 0.006781 | 0.048549 |
| ENSG00000116774 | 14.61802 | 4.226313 | 3.458812 | 0.00671 | 0.04867 |
| ENSG00000116285 | 10.81474 | 29.14543 | 0.371061 | 0.00688 | 0.049097 |
| ENSG00000184730 | 3819.081 | 1196.414 | 3.192106 | 0.006909 | 0.049142 |
| ENSG00000112306 | 9161.323 | 3949.685 | 2.319507 | 0.006977 | 0.049468 |

Table S3 The four positive lncRNAs and their targets of predicted mRNAs

Four lncRNAs were predicted with mRNA targets related to 32 genes. A total of 16 genes were included in the network of MSTRG.63013, with a correlation score of more than 0.9; and there were 8 genes within 50 kb of MSTRG.63013

| lncRNA | Gene | Fold Change | Up/Down | Gene Name | Position |
| --- | --- | --- | --- | --- | --- |
| MSTRG.166799 | ENSG00000059378* | 1.116477 | up | PARP12 | chr7:140023744-140063721:- |
| MSTRG.72098 | ENSG00000121073* | 0.9905519 | down | SLC35B1 | chr17:49700943-49709014:- |
| MSTRG.72098 | ENSG00000121104* | 1.0051937 | up | FAM117A | chr17:49710332-49789180:- |
| MSTRG.72098 | ENSG00000121067* | 1.0993232 | up | SPOP | chr17:49598884-49678234:- |
| MSTRG.63013 | ENSG00000168724# | 0.689054871 | down | DNAJC21 | chr5:34929593-34958964:+ |
| MSTRG.63013 | ENSG00000102753# | 0.655152925 | down | KPNA3 | chr13:49699307-49792921:- |
| MSTRG.63013 | ENSG00000168385# | 0.772103492 | down | SEPT2 | chr2:241315100-241354027:+ |
| MSTRG.63013 | ENSG00000137492# | 0.750963221 | down | THAP12 | chr11:76349956-76380971:- |
| MSTRG.63013 | ENSG00000115233# | 0.645640356 | down | PSMD14 | chr2:161308038-161411717:+ |
| MSTRG.63013 | ENSG00000136758# | 0.867647392 | down | YME1L1 | chr10:27110112-27155266:- |
| MSTRG.63013 | ENSG00000067248# | 0.646261578 | down | DHX29 | chr5:55256245-55307722:- |
| MSTRG.63013 | ENSG00000137040# | 0.668202891 | down | RANBP6 | chr9:6011043-6015625:- |
| MSTRG.63013 | ENSG00000138757# | 0.71776591 | down | G3BP2 | chr4:75642782-75724525:- |
| MSTRG.63013 | ENSG00000122068# | 0.711134446 | down | FYTTD1 | chr3:197737179-197787596:+ |
| MSTRG.63013 | ENSG00000106591# | 0.664334611 | down | MRPL32 | chr7:42932200-42948958:+ |
| MSTRG.63013 | ENSG00000122958# | 0.79703837 | down | VPS26A | chr10:69123512-69172861:+ |
| MSTRG.63013 | ENSG00000163320# | 0.676867453 | down | CGGBP1 | chr3:88051944-88149885:- |
| MSTRG.63013 | ENSG00000065150# | 0.710456436 | down | IPO5 | chr13:97953658-98024297:+ |
| MSTRG.63013 | ENSG00000172115# | 0.504225165 | down | CYCS | chr7:25120091-25125361:- |
| MSTRG.63013 | ENSG00000004700# | 0.736786503 | down | RECQL | chr12:21468911-21501669:- |
| MSTRG.63013 | ENSG00000162069* | 11.14552942 | up | BICDL2 | chr16:3027682-3036926:- |
| MSTRG.63013 | ENSG00000131652* | 0.941984198 | down | THOC6 | chr16:3024027-3027755:+ |
| MSTRG.63013 | ENSG00000103145* | 1.212027078 | up | HCFC1R1 | chr16:3022620-3024286:- |
| MSTRG.63013 | ENSG00000006327* | 5.716030562 | up | TNFRSF12A | chr16:3018445-3022383:+ |
| MSTRG.63013 | ENSG00000008517* | 0.864043072 | down | IL32 | chr16:3065297-3082192:+ |
| MSTRG.63013 | ENSG00000213937* | 4.511953792 | up | CLDN9 | chr16:3012456-3014505:+ |
| MSTRG.63013 | ENSG00000270168* | / | up | LA16c-380H5.3 | chr16:2988256-3002016:+ |
| MSTRG.63013 | ENSG00000130182* | 1.300184891 | up | ZSCAN10 | chr16:3088890-3099317:- |
| ENSG00000267174 | ENSG00000105518* | 1.4473762 | up | TMEM205 | chr19:11342776-11346518:- |
| ENSG00000267174 | ENSG00000130158* | 1.02462 | up | DOCK6 | chr19:11199295-11262481:- |
| ENSG00000267174 | ENSG00000183401* | 1.0834158 | up | CCDC159 | chr19:11344684-11354944:+ |
| ENSG00000267174 | ENSG00000105520* | 1.9968091 | up | PLPPR2 | chr19:11355386-11365698:+ |

“#” means the gene is correlated with the lncRNA; “*” means the gene is within 50kb of the lncRNA.
